# Supplementary material for: Computational Mechanisms of Approach-Avoidance Conflict Predictively Differentiate Between Affective and Substance Use Disorders
Source: Comput Psychiatr. 2025 Sep 5;9(1):159–86. doi: 10.5334/cpsy.131 (PMC12421128; doi:10.5334/cpsy.131)
Supplement: Supplementary Materials. — Supplementary Results, Figures, and Tables. [file cpsy-9-1-131-s1.pdf]

Supplementary Materials

Computational mechanisms of approach-avoidance conflict predictively differentiate between affective and substance use disorders

Marishka M. Mehta[1,2], Navid Hakimi[1], Orestes Pena[1], Taylor Torres[1], Carter M. Goldman[1], Claire A. Lavalley[1,2], Jennifer L. Stewart[1], Hannah Berg[1], Maria Ironside[1], Martin P. Paulus[1], Robin Aupperle[1], Ryan Smith[1*]

[1]Laureate Institute for Brain Research, Tulsa, OK, United States

[2]University of Tulsa, Tulsa, OK, United States

Table of Contents

Intra-class correlations **1**

Model parameters by group 1

Descriptive task measures 1

Intra-class correlations for post-task survey questions **5**

Relationship between model parameters and other measures at follow-up **5**

Demographic variables 5

Descriptive task measures 6

Post-task surveys 6

Group differences in model parameters when only including participants who returned for follow-up **7**

Group differences in post-task self-report questionnaire ratings **12**

Group differences in the relationship between response times and chosen runway positions **13**

Group differences in the relationship between response times and model parameters **14**

Supplementary Figures **16**

Supplementary Figure 1 16

Supplementary Figure 2 18

Supplementary Figure 3 19

Supplementary Figure 4 20

Supplementary Figure 5 21

Supplementary Figure 6 22

Supplementary Figure 7 23

Supplementary Figure 8 24

Supplementary Figure 9 25

Supplementary Figure 10 25

Supplementary Tables **26**

Supplementary Table 1. Comorbidity table for participants who returned for follow-up 26

Supplementary Table 2. Differences in participant characteristics at baseline for those who did vs. did not return for follow-up 26

Supplementary Table 3. Baseline symptom and demographic characteristics by group for participants who did vs. did not return for follow-up 26

Supplementary Table 4. Group-wise differences in participant characteristics at 1-year follow-up in the exploratory and confirmatory sample ... 27

Supplementary Table 5. Group-wise intra-class correlations for clinical measures at baseline and 1-year follow-up ... 27

Supplementary Table 6. Group-wise intra-class correlations for post-task self-report questionnaire items at baseline and 1-year follow-up ... 29

Supplementary Table 7. Group-wise post-task self-report questionnaire items at baseline and 1-year follow-up, and correlations with computational model parameters at follow-up ... 30

Supplementary Table 8. Results of linear mixed effects models predicting *DU* and *EC* in data including all baseline participants, when accounting for effects of group and time ... 32

Supplementary Table 9. Results of linear mixed effects models predicting *DU* and *EC* in data including all baseline participants, when accounting for effects of group, time, age, sex, and WRAT scores ... 32

Supplementary Table 10. Summary statistics for response times (Mean (SD)) at baseline and follow-up ... 34

Supplementary Table 11. Summary statistics for average chosen runway position (Mean (SD)) at baseline and follow-up ... 35

Supplementary Table 12. Summary statistics for variability (SD) in chosen runway position (Mean (SD)) at baseline and follow-up ... 36

Supplementary Table 13. Results of linear mixed effects models predicting response times (RTs) in participants who returned for follow-up, when accounting for effects of group, time, and their interaction ... 37

Supplementary Table 14. Results of linear mixed effects models predicting response times (RTs) in participants who returned for follow-up, when accounting for effects of group, time, age, and sex .. 38

Supplementary Table 15. Results of linear mixed effects models predicting response times (RTs) in participants who returned for follow-up, when accounting for effects of group, time, age, and sex .. 39

Supplementary Table 16. Results of linear mixed effects models predicting average chosen runway position in participants who returned for follow-up, when accounting for effects of group, time, and their interaction ... 42

Supplementary Table 17. Results of linear mixed effects models predicting average chosen runway position in participants who returned for follow-up, when accounting for effects of group, time, age, and sex ... 43

Supplementary Table 18. Results of linear mixed effects models predicting average chosen runway position in participants who returned for follow-up, when accounting for effects of group, time, age, sex, and WRAT scores ... 45

Supplementary Table 19. Results of linear mixed effects models predicting choice variability in participants who returned for follow-up, when accounting for effects of group, time, and their interaction ... 47

Supplementary Table 20. Results of linear mixed effects models predicting choice variability in participants who returned for follow-up, when accounting for effects of group, time, age, and sex .. 49

Supplementary Table 21. Results of linear mixed effects models predicting choice variability in participants who returned for follow-up, when accounting for effects of group, time, age, sex and WRAT scores 51

Supplementary Table 22. Results of linear mixed effects models predicting self-report questionnaire items in participants who returned for follow-up, when accounting for effects of group, time, and their interaction 53

Supplementary Table 23. Results of linear mixed effects models predicting self-report questionnaire items in participants who returned for follow-up, when accounting for effects of group, time, age, and sex 54

Supplementary Table 24. Results of linear mixed effects models predicting self-report questionnaire items in participants who returned for follow-up, when accounting for effects of group, time age, sex, and WRAT scores 55

Supplementary Table 25. Summary statistics for model parameters (Mean (SD)) for each sub-diagnosis at baseline and follow-up in the exploratory and the confirmatory samples 57

Supplementary Table 26. Results of linear mixed effects models predicting *DU* when accounting for effects of group, time, and their interaction 58

Supplementary Table 27. Results of linear mixed effects models predicting *DU* when accounting for effects of group, time, age, and sex 59

Supplementary Table 28. Results of linear mixed effects models predicting *EC* when accounting for effects of group, time, and their interaction 62

Supplementary Table 29. Results of linear mixed effects models predicting *EC* when accounting for effects of group, time, age, and sex 64

Supplementary Table 30. Simulation-based post-hoc power analyses to detect group differences between HCs and clinical subgroups 69

Supplementary Table 31. Performance metrics for predictive categorization of individuals with affective disorders (no comorbid SUDs) and those with SUDs (but no comorbid affective disorders) 69

Supplementary Table 32. Performance metrics for predictive categorization of individuals with and without SUDs 70

Supplementary Table 33. Performance metrics for predictive categorization of individuals with and without affective disorders 71

Supplementary Table 34. Performance metrics for predictive categorization of individuals with and without comorbid affective and substance use disorders 71

Intra-class correlations (ICCs) in model parameters by group

ICCs for *DU* by group were as follows:

HCs: ICC = 0.66 ($F(68,68) = 4.81$, $p < 0.001$)

DEP/ANX: ICC = 0.61 ($F(134,134) = 4.10$, $p < 0.001$)

SUDs: ICC = 0.44 ($F(82,82) = 2.62$, $p < 0.001$)

ICCs for *EC* by group were as follows:

HCs: ICC = 0.76 ($F(68,68) = 7.16$, $p < 0.001$)

DEP/ANX: ICC = 0.70 ($F(134,134) = 5.78$, $p < 0.001$)

SUDs: ICC = 0.45 ($F(82,82) = 2.64$, $p < 0.001$)

Intra-class correlations in descriptive task measures

For all model-free task measures, ICCs were first calculated across all participants and then by group for the five trial types.

Response time

ICCs for all participants were as follows:

All trials: ICC = 0.56 ($F(285,285) = 3.52$, $p < 0.001$)

AV: ICC = 0.50 ($F(284,284) = 2.98$, $p < 0.001$)

APP: ICC = 0.51 ($F(285,285) = 3.05$, $p < 0.001$)

CONF2: ICC = 0.49 ($F(285,285) = 2.91$, $p < 0.001$)

CONF4: ICC = 0.51 ($F(285,285) = 3.09$, $p < 0.001$)

CONF6: ICC = 0.43 ($F(285,285) = 2.54$, $p < 0.001$)

ICCs for HCs were as follows:

All trials: ICC = 0.62 ($F(68,68) = 4.28$, $p < 0.001$)

AV: ICC = 0.62 ($F(68,68) = 4.25$, $p < 0.001$)

APP: ICC = 0.54 ($F(68,68) = 3.35$, $p < 0.001$)

CONF2: ICC = 0.47 ($F(68,68) = 2.77$, $p < 0.001$)

CONF4: ICC = 0.55 ($F(68,68) = 3.47$, $p < 0.001$)

CONF6: ICC = 0.51 ($F(68,68) = 2.05$, $p < 0.001$)

ICCs for DEP/ANX were as follows:

All trials: ICC = 0.59 ($F(133,133) = 3.88$, $p < 0.001$)

AV: ICC = 0.47 ($F(133,133) = 2.75$, $p < 0.001$)

APP: ICC = 0.53 ($F(133,133) = 3.22$, $p < 0.001$)

CONF2: ICC = 0.54 ($F(133,133) = 3.32$, $p < 0.001$)

CONF4: ICC = 0.52 ($F(133,133) = 3.20$, $p < 0.001$)

CONF6: ICC = 0.50 ($F(133,133) = 3.03$, $p < 0.001$)

ICCs for SUDs were as follows:

All trials: ICC = 0.48 ($F(82,82) = 2.81$, $p < 0.001$)

AV: ICC = 0.36 ($F(82,82) = 2.11$, $p < 0.001$)

APP: ICC = 0.46 ($F(82,82) = 2.72$, $p < 0.001$)

CONF2: ICC = 0.44 ($F(82,82)= 2.60$, $p < 0.001$)

CONF4: ICC = 0.47 ($F(82,82) = 2.78$, $p < 0.001$)

CONF6: ICC = 0.32 ($F(82,82) = 1.93$, $p = 0.002$)

Average chosen runway position

ICCs for all participants were as follows:

All trials: ICC = 0.70 ($F(286,286) = 5.61$, $p < 0.001$)

AV: ICC = 0.62 ($F(286,286) = 4.24$, $p < 0.001$)

APP: ICC = 0.54 ($F(286,286) = 3.36$, $p < 0.001$)

CONF2: ICC = 0.69 ($F(286,286) = 5.53$, $p < 0.001$)

CONF4: ICC = 0.70 ($F(286,286) = 5.55$, $p < 0.001$)

CONF6: ICC = 0.71 ($F(286,286) = 5.95$, $p < 0.001$)

ICCs for HCs were as follows:

All trials: ICC = 0.84 ($F(68,68) = 11.44$, $p < 0.001$)

AV: ICC = 0.80 ($F(68,68) = 9.17$, $p < 0.001$)

APP: ICC = 0.50 ($F(68,68) = 3.03$, $p < 0.001$)

CONF2: ICC = 0.80 ($F(68,68) = 9.01$, $p < 0.001$)

CONF4: ICC = 0.82 ($F(68,68) = 10.04$, $p < 0.001$)

CONF6: ICC = 0.88 ($F(68,68) = 15.03$, $p < 0.001$)

ICCs for DEP/ANX were as follows:

All trials: ICC = 0.70 ($F(134,134) = 5.69$, $p < 0.001$)

AV: ICC = 0.56 ($F(134,134) = 3.59$, $p < 0.001$)

APP: ICC = 0.45 ($F(134,134) = 2.66$, $p < 0.001$)

CONF2: ICC = 0.71 ($F(134,134) = 5.85$, $p < 0.001$)

CONF4: ICC = 0.69 ($F(134,134) = 5.39$, $p < 0.001$)

CONF6: ICC = 0.67 ($F(134,134) = 5.13$, $p < 0.001$)

ICCs for SUDs were as follows:

All trials: ICC = 0.43 ($F(82,82) = 2.50$, $p < 0.001$)

AV: ICC = 0.53 ($F(82,82) = 3.25$, $p < 0.001$)

APP: ICC = 0.69 ($F(82,82) = 5.49$, $p < 0.001$)

CONF2: ICC = 0.33 ($F(82,82)= 1.97$, $p = 0.001$)

CONF4: ICC = 0.30 ($F(82,82) = 1.85$, $p = 0.003$)

CONF6: ICC = 0.35 ($F(82,82) = 2.05$, $p < 0.001$)

Choice variability

ICCs for all participants were as follows:

All trials: ICC = 0.62 ($F(286,286) = 4.22$, $p < 0.001$)

AV: ICC = 0.51 ($F(286,286) = 3.06$, $p < 0.001$)

APP: ICC = 0.39 ($F(286,286) = 2.25$, $p < 0.001$)

CONF2: ICC = 0.37 ($F(286,286) = 2.17$, $p < 0.001$)

CONF4: ICC = 0.38 ($F(286,286) = 2.20$, $p < 0.001$)

CONF6: ICC = 0.37 ($F(286,286) = 2.20$, $p < 0.001$)

ICCs for HCs were as follows:

All trials: ICC = 0.77 ($F(68,68) = 7.78$, $p < 0.001$)

AV: ICC = 0.71 ($F(68,68) = 5.95$, $p < 0.001$)

APP: ICC = 0.58 ($F(68,68) = 3.80$, $p < 0.001$)

CONF2: ICC = 0.47 ($F(68,68) = 2.78$, $p < 0.001$)

CONF4: ICC = 0.52 ($F(68,68) = 3.20$, $p < 0.001$)

CONF6: ICC = 0.34 ($F(68,68) = 2.04$, $p = 0.002$)

ICCs for DEP/ANX were as follows:

All trials: ICC = 0.66 ($F(134,134) = 4.85$, $p < 0.001$)

AV: ICC = 0.47 ($F(134,134) = 2.74$, $p < 0.001$)

APP: ICC = 0.29 (F(134,134) = 1.80, p < 0.001)

CONF2: ICC = 0.50 (F(134,134) = 2.96, p < 0.001)

CONF4: ICC = 0.28 (F(134,134) = 1.79, p < 0.001)

CONF6: ICC = 0.44 (F(134,134) = 2.56, p < 0.001)

ICCs for SUDs were as follows:

All trials: ICC = 0.30 (F(82,82) = 1.86, p = 0.003)

AV: ICC = 0.34 (F(82,82) = 2.03, p < 0.001)

APP: ICC = 0.37 (F(82,82) = 2.16, p < 0.001)

CONF2: ICC = 0.12 (F(82,82)= 1.27, p = 0.141)

CONF4: ICC = 0.36 (F(82,82) = 2.14, p = 0.003)

CONF6: ICC = 0.33 (F(82,82) = 1.97, p = 0.001)

Intra-class correlations for post-task survey questions

ICCs for post-task survey questions are provided in **Supplementary Table 6**. Across items, the reliability was poor to good for HCs (ICCs between 0.33 and 0.68) and fair to good for DEP/ANX (ICCs between 0.45 and 0.68). In SUDs, poor to fair reliability (ICCs between 0.31 and 0.53) was found for most post-task survey questions. However, ICCs for questions about the basic approach (Q4; ICC = 0.17, p=0.06) and avoidance (Q5; ICC = 0.12, p = 0.15) motivations were statistically non-significant.

Relationship between model parameters and demographic variables at follow-up

Similar to our findings in the *exploratory* sample and the *confirmatory* baseline sample, no sex differences were found in *EC* (t(131.75) = 0.86, p = 0.39) or *DU* (t(125.24) = -0.06, p = 0.95) at 1-year follow-up. *DU* was also positively correlated with age (r = 0.14, p =0.017). We failed to

replicate the relationship between the *EC* parameter and age ($r = 0.04$, $p = 0.51$).

Relationship between model parameters and descriptive task measures at follow-up

We replicated the relationship between model parameters and RTs and choice variability (see **Supplementary Figure 2**). In addition, as we saw in the baseline sample, there was a positive correlation between *DU* and APP RTs ($r = 0.51$, $p < 0.001$) along with *EC* and variability in runway choices in APP condition ($r = 0.12$, p=0.35). The relationships between model parameters and the average chosen runway position were identical to those found at baseline (see **Supplementary Figure 2** for details).

Relationship between model parameters and post-task questionnaire ratings at follow-up

Here we report group-wise differences in post-task survey responses and their correlation with model parameters. The relationship between *EC* and post-task surveys was preserved in HCs and DEP/ANX. In individuals with SUDs, the correlation between *EC* and using distraction (Q7; $r = 0.3$, $p = 0.07$) and self-regulation (Q8; $r = 0.09$, $p = 0.39$) strategies during the presentation of aversive images were not statistically significant. For the within-group correlation of *DU* and post-task surveys, the relationships observed across participants were also seen in individuals with DEP/ANX. Relationships between *DU* and avoidance of negative stimuli (Q5; HCs: $r = 0.13$, $p = 0.31$), and between *DU* and keeping eyes open during presentation of negative stimuli (Q6; HCs: $r = -0.11$ $p = 0.39$; SUDs: $r = -0.12$, $p = 0.30$) were not statistically significant. The full results of the correlational analyses between model parameters and items in the post-task survey (Q1-Q8) are shown in **Supplementary Table 7**. Additional reports of specific relationships found significant at baseline are visualized in **Supplementary Figure 3**.

Group differences in model parameters when only including participants who returned for follow-up

We perform LMEs identical to those in the main text in a sub-sample of participants who returned for the follow-up visit. The first set of LMEs examines the main effects of the group, time, and their interaction. Additional LMEs include the effects of age, sex, and their interactions with the group, along with the effects of group and time. Lastly, LMEs accounting for WRAT scores were also performed.

Similar to the results in the main text, there was a main effect of time ($F(1, 283) = 21.46$, $p < 0.001$) on *DU*, suggesting a reduction in *DU* over time. In an LME which also includes the effects of age and sex, the main effect of time was conserved ($F(1, 292) = 26.39$, $p < 0.001$), and a main effect of age was observed ($F(1, 279) = 7.75$, $p < 0.01$), suggesting that *DU* may increase with age. These effects were conserved when accounting for WRAT scores. A main effect of WRAT scores ($F(1, 232) = 13.69$, $p<0.001$) was also found. No other statistically significant effects or interactions were found.

When predicting *EC*, a main effect of group was observed ($F(2, 283) = 20.29$, $p < 0.001$), where SUDs (EMM = 0.37) displayed lower *EC* than DEP/ANX (EMM = 0.819; $t(279)=4.645$, $p < 0.001$, $d=1.02$) and HCs (EMM = 1.065; $t(284)=6.158$, $p < 0.001$, $d=1.58$). HCs displayed greater *EC* than DEP/ANX ($t(284)=-2.402$, $p = 0.017$, $d=-0.56$). In the follow-up LME, the main effect of group ($F(2, 278) = 13.49$, $p < 0.001$) was conserved, even when controlling for WRAT scores ($F(2, 232) = 10.83$, $p < 0.001$). A *group x WRAT* interaction ($F(2, 233) = 4.59$, $p = 0.011$) was also found. No other significant effects were observed.

As in our baseline study, we repeated the LMEs in female-only (N=213) and male-only (N=74) samples. The LMEs accounted for the effect of group, age (its interaction with group), and time. As expected, there was a main effect of group on *EC* ($F(2,207.28) = 18.46$, $p < 0.001$) in

females, but not in males ($F(2,68) = 2.33$, $p = 0.105$; see **Supplementary Figure 5**). Post-hoc contrasts revealed females in the DEP/ANX (EMM=0.807; $t(207.01)$=-3,125, $p < 0.01$, d = -0.84) and SUDs (EMM = 0.351;$t(207.03)$=6.23, $p < 0.001$, d = 1.86) groups had lower *EC* values than HCs (EMM = 1.184), with the lowest value for SUDs ($t(207.04)$=4.14, $p < 0.001$, d=1.02). No other significant effects were found.

Analysis of descriptive task measures including all baseline participants

The descriptive statistics and group differences in task performance measures are provided in **Supplementary Tables 10-12**. Distributions of the average chosen runway position during the longitudinal visit are shown in **Supplementary Figure 6.** An LME was also performed to evaluate the effect of group, time, and their interaction. Follow-up LMEs were performed to account for the effects of age, sex, and their interaction with group. A summary of the replication of these results is provided in **Supplementary Figure 7**. Similar to our baseline paper, subsequent LME also accounted for the effect of the WRAT score and its interaction with group. Analogous analyses were also performed in a sub-sample of participants, where the baseline data was limited to participants who returned for their follow-up visit. These results are described below. The LMEs were also performed in a sub-sample of participants who returned for the follow-up visit (see **Supplementary Tables 13-21)**.

Response times

When considering all trials, there was a main effect of time ($F(1, 340) = 11.33$, $p < 0.001$), whereby participants displayed faster RTs at 1-year follow-up. This was also seen in APP, CONF2, CONF4, and CONF6 trials ($ts \geq 2.55$, $ps \leq 0.014$). A main effect of group was observed in AV trails ($F(2, 487) = 14.13$, $p < 0.001$), where SUDs (EMM = 1.489) showed slower RTs than both HCs (EMM = 1.303; $t(462.37)$=-4.751, $p < 0.001$, d = -0.75) and DEP/ANX (EMM =

1.332; $t(479.75)=-4.902$, $p < 0.001$, $d = -0.63$). No other effects of group, time, or their interaction were found.

Next, we accounted for possible effects of age and sex. Across all trials, the main effect of time was conserved ($F(1, 356.08) = 20.40$, $p < 0.001$); a positive association with age was also present ($F(1, 459.06) = 97.16$, $p < 0.001$). When restricting to specific trial types, there was a main effect of the group in AV ($F(2, 465) = 14.29$, $p < 0.001$) and APP ($F(2, 466) = 3.22$, $p = 0.041$) trials. During AV trials, SUDs (EMM = 1.491) had slower response times than HCs (EMM = 1.326, $t(442.01)=-5.389$, $p < 0.001$, $d=-0.78$) and DEP/ANX (EMM = 1.326, $t(468.92)=-5.671$, $p < 0.001$, $d=-0.67$). In the APP condition, DEP/ANX (EMM = 1.187) had faster response times than SUDs (EMM = 1.255; $t(470.06)=-2.216$, $p = 0.027$, $d=-0.28$). A *group x sex* interaction ($F(2, 467) = 7.64$, $p < 0.001$) was present in the AV trials. In females, SUDs (EMM = 1.51) displayed the highest RTs, followed by DEP/ANX (EMM = 1.338) and HCs (EMM = 1.237; $ts \geq 2.43$, $ps \leq 0.016$). In males, DEP/ANX (EMM = 1.297) were significantly faster than HCs (EMM = 1.442; $t(457.87)=-2.415$, $p = 0.016$, $d=-0.59$) and SUDs (EMM = 1.448; $t(506.08)=-2.792$, $p = 0.005$, $d=-0.61$). Significant effects of age (Fs ≥ 55.94, ps < 0.001) and time ($Fs \geq 9.18$, $ps \leq 0.003$) were observed within all trial types. No other significant main effects or interactions were observed. When accounting for the effect of WRAT scores, a significant *group x WRAT* interaction was found in all conflict trials and when all trials were considered together ($Fs > 3.41$, $ps \leq 0.034$). There was a main effect of WRAT (Fs > 5.90, $ps < 0.016$) in all comparisons except for the CONF2 and CONF4 trials. In addition, a main effect of sex ($F(1, 381) = 4.64$, $p = 0.032$) was also present in AV trials, such that males had slower response times than females.

Chosen Runway Position

There was a main effect of group when all trials were considered and for each trial type (*Fs* $\geq$ 4.73, *ps* $\leq$ 0.009), except for APP trials ($F(2, 494) = 1.22$, $p = 0.295$). Post-hoc contrast revealed that SUDs consistently displayed lower avoidance than HCs ($|ts| \geq 4.34$, $ps < 0.001$) and DEP/ANX ($|ts| \geq 3.19$, $ps \leq 0.002$) in the AV and CONF conditions.

Notably, there was also a main effect of time in APP ($F(1, 334) = 5.91$, $p = 0.016$) and AV trials ($F(1, 336) = 11.16$, $p < 0.001$), indicating a greater drive to approach the points ($EMM_{Baseline}$ = 8.183; $EMM_{Follow\text{-}up}$ = 8.396) and avoid the aversive image ($EMM_{Baseline}$ = 7.648; $EMM_{Follow\text{-}up}$ = 7.998) in the absence of conflict at 1-year follow-up. A *group x time* interaction was found in AV trials ($F(2, 337) = 3.60$, $p = 0.028$), whereby SUDs ($EMM_{Baseline}$ = 6.782; $EMM_{Follow\text{-}up}$ = 7.453) at both time points displayed lower avoidance than HCs (Baseline: EMM = 8.249, $t(615.99)=6.568$, $p < 0.001$, d=1.29; Follow-up: EMM = 8.27; $t(754.42)=3.055$, $p = 0.002$, d = 0.72) and DEP/ANX (Baseline: EMM = 8.009, $t(615.99)=6.78$, $p < 0.001$, $d = 1.08$; Follow-up: EMM = 8.267; $t(760.71)=3.601$, $p < 0.001$, d=0.72). No other significant main effects or interactions were observed.

The majority of these results were conserved when effects of age, sex, and their interactions with the group were included (*Fs* $\geq$ 7.59, *ps* < 0.001). There was a main effect of sex when all trials were considered ($F(1, 472) = 4.66$, $p = 0.031$) and in all CONF conditions (*Fs* $\geq$ 4.10, *ps* $\leq$ 0.043), whereby males displayed lower avoidance than females. *Group x sex* interaction was seen in CONF2 and CONF4 conditions, where we see the conservation of group effects in females ($|ts| \geq 2.942$, $ps \leq 0.003$) but not in males. The main effect of age was also present in all conditions (*Fs* $\geq$ 6.59, *ps* $\leq$ 0.008). Lastly, when controlling for the effects of the WRAT score and its interaction with group, *group x sex* interaction was found in CONF6 trials ($F(2, 382)$ = 3.11, p<0.05). There was main effect of WRAT in APP ($F(1, 364) = 15.78$, p<0.001) and AV

($F(1, 360) = 26.05$, $p<0.001$) trials and *group x WRAT* interaction in CONF2 trials ($F(2, 371) = 3.82$, $p = 0.023$).

Choice Variability

As expected, variance in chosen runway position was lower at follow-up compared to baseline when all trials were considered and within each trial type (*Fs* < 5.90, *ps* ≤ 0.016). SUDs ($EMM_{APP} = 1.006$; $EMM_{AV} = 1.543$) also displayed higher choice variance than HCs ($EMM_{APP} = 0.605$; $EMM_{AV} = 0.799$) and DEP/ANX ($EMM_{APP} = 0.708$; $EMM_{AV} = 0.844$) group for the AV and APP trials (|*ts*| > 2.81, *ps* ≤ 0.005). We failed to replicate the group differences ($F(2, 481) = 0.66$, $p = 0.518$) in the CONF6 trial, which were also absent at baseline. There was also a *group x time* interaction in AV trials ($F(2, 352) = 3.48$, $p = 0.032$), indicating that SUDs ($EMM_{Baseline} = 1.755$; $EMM_{Follow\text{-}up} = 1.189$) also had higher choice inconsistencies than HCs ($EMM_{Baseline} = 0.831$; $EMM_{Follow\text{-}up} = 0.746$) and DEP/ANX ($EMM_{Baseline} = 0.949$; $EMM_{Follow\text{-}up} = 0.668$) at baseline and follow-up (|*ts*| < 2.40, *ps* ≤ 0.017). These results were conserved when effects of age, sex, and their interactions with group were included (*Fs* < 6.62, *ps* ≤ 0.001). When all trials were considered, there was a main effect of sex ($F(1, 471) = 4.33$, $p = 0.038$), whereby females (EMM = 2.01) displayed greater variability in their choices than males (EMM = 1.79). There was also a main effect of age (*Fs* ≥ 13.52, *ps* < 0.001) for each trial type and when all trials were considered ($F(1, 463) = 13.63$, $p < 0.001$). A *group x* age interaction (*Fs* ≥ 3.96, *ps* ≤ 0.02) was found for all CONF trials, and a main effect of group was also found for CONF4 trials ($F(2, 464) = 3.91$, $p = 0.021$). Lastly, additional LMEs were performed to account for the effects of WRAT score and its interaction with group. A main effect of WRAT scores was found in each individual trial type (*Fs* ≥ 12.57, *ps* < 0.001). Once included, the *group x age* interaction in CONF trials (*Fs*

≤ 2.64, *ps*$\geq$0.073) and group effect in APP trials ($F(2, 372) = 0.45$, $p = 0.641$) were no longer significant.

Group differences in post-task self-report questionnaire ratings

Additional LMEs were performed to assess differences in select self-report questionnaire items (**Table 5**, main text) measuring anxiety (Q2), difficulty in decision-making during the task (Q3), approach motivation (Q4) and avoid motivation (Q5). A summary of the replication of these results is provided in **Supplementary Figure 8**. Analogous analyses were also performed in a sub-sample of participants, where the baseline data was limited to participants who returned for their follow-up visit. These results are provided in **Supplementary Tables 22-24**.

For self-reported anxiety (Q2), a main effect of group ($F(2, 489) = 3.99$, $p = 0.019$) was observed. SUDs (EMM = 3.72) displayed lower self-reported anxiety than DEP/ANX (EMM = 4.22; $t(480.34) = 2.685$, $p = 0.007$, $d = 0.37$). When effects of age and sex were considered, sex effect ($F(1, 468) = 11.57$, $p < 0.001$) and *group x sex* interaction ($F(2, 469) = 5.75$, $p < 0.001$) better explained the group effects ($F(2, 273) = 1.06$, $p = 0.349$). In female participants SUDs (EMM = 3.68) reported lower anxiety than DEP/ANX (EMM = 4.36; $t(457.41)=3.103$, $p = 0.002$, $d = 0.51$) and HCs (EMM = 4.52, $t(438.16)=2.988$, $p = 0.003$, $d = 0.62$). No significant group effects were found in males. No other main effects or interactions were found. However, when accounting for WRAT scores, only a main effect of sex was found ($F(1, 382) = 11.08$, $p < 0.001$). Females (EMM =4.15) reported greater anxiety ($t(387.68)=3.115$, $p = 0.002$, $d = 0.51$) than males (EMM = 3.46).

For self-reported difficulty in decision-making during the task (Q3), there was a main effect of time ($F(1, 363) = 4.72$, $p = 0.03$), suggesting participants had less difficulty making decisions during the task at follow-up ($EMM_{Baseline} = 2.31$; $EMM_{Follow\text{-}up} = 2.10$; $t(367.49) = 2.14$, $p < 0.05$,

d = 0.17). When the effects of age and sex were considered, the main effect of time ($F(1, 368)$ = 5.51, $p = 0.019$) persisted. In addition, a main effect of age ($F(1, 455) = 6.63$, $p = 0.010$) and *group x age* interaction was found ($F(2, 457) = 4.55$, $p = 0.011$). No additional main effects or interactions were found. However, when accounting for WRAT scores, no significant main effects or interactions were found ($Fs \leq 3.38$, $ps \geq 0.067$).

For self-reported approach motivation (Q4), main effect of group ($F(2, 492) = 6.84$, $p < 0.001$) was observed. SUDs (EMM = 5.40) displayed lower self-reported approach motivation than DEP/ANX (EMM = 4.76; $t(482.32)$=-2.807, $p = 0.005$, d = -0.41) and HCs (EMM = 4.41; $t(467.38)$=-3.598, $p < 0.001$, d = -0.65). These effects ($F(2, 468) = 4.87$, $p = 0.008$) were conserved after accounting for the effects of age and sex. A main effect of age was also found ($F(1, 461) = 5.67$, $p = 0.018$). No other main effects or interactions were found. Identical results were found when accounting for the effects of WRAT scores.

For self-reported avoid motivation (Q5), main effect of group ($F(2, 488) = 6.27$, $p = 0.002$) was observed. SUDs (EMM = 2.41) displayed lower self-reported avoidance motivation than DEP/ANX (EMM = 3.08; $t(479.91)$=3.248, $p = 0.001$, $d = 0.44$) and HCs (EMM = 3.24; $t(463.81)$=3.29, $p = 0.001$, d = -0.54). These effects ($F(2, 467) = 4.18$, $p = 0.016$) were conserved after accounting for the effects of age and sex. A *group x sex* interaction ($F(2, 469) = 4.38$, p = 0.014) was found, where these group differences (Females: EMM_{HCs} = 3.64; $EMM_{DEP/ANX}$ = 3.14, EMM_{SUDs} = 2.204; Male: EMM_{HCs} = 2.56; $EMM_{DEP/ANX}$ = 2.90; EMM_{SUDs} = 2.67) were driven by female participants (DEP/ANX - SUDs: $t(457.54)$=3.717, $p < 0.001$, $d = 0.61$; HCs - SUDs: $t(438.38)$=4.489, $p < 0.001$, $d = 0.93$). However, when we controlled for WRAT scores, the group effect ($F(2, 375) = 4.41$, $p = 0.013$) was conserved, and the *group x sex* interaction

($F(2, 381) = 2.99$, p=0.052) was marginal. In addition, a main effect of age ($F(1, 365) = 4.69$, p = 0.031) was found.

Group differences in the relationship between response times and chosen runway positions

An LME was performed to examine potential group differences in the relationship between RTs and chosen runway position in the combined exploratory and confirmatory sample: *chosen runway position ~ group + time + RTs + group*time + group*RTs + group*time*RTs*. There was a main effect of group ($F(2, 936.06) = 10.93$, $p < 0.001$), reflecting greater approach behavior in DEP/ANX (EMM = 7.23; $t(915) = -2.52$, $p = 0.025$) and SUDs (EMM = 7.58; $t(946) = -4.46$, $p < 0.001$) than in HCs (EMM = 6.91) overall. SUDs also had greater approach behavior than DEP/ANX ($t(977) = -3.14$, $p = 0.002$). An overall negative relationship was found between RTs and chosen runway position ($F(1, 1553.42) = 141.32$, $p < 0.001$). This LME also revealed a *group x RT* interaction ($F(2, 1547.32) = 4.78$, $p = 0.009$), suggesting a stronger negative association between RTs and approach behavior in SUDs (ET = -1.94) than in HCs (ET = -0.59; $t(1533) = 3.13$, $p = 0.005$). Lastly, there was a *group x time* x *RT* interaction ($F(3, 831.67) = 3.43$, $p = 0.017$). This interaction was due to a decrease in the magnitude of the inverse relationship between RTs and runway position over time in the DEP/ANX group ($ET_{Baseline} = -1.75$; $ET_{Follow\text{-}up} = -0.93$; $t(812) = -2.95$, $p = 0.003$), while the relationship between RTs and runway position remained relatively stable over time in the other two groups (HC: $ET_{Baseline} = -0.91$; $ET_{Follow\text{-}up} = -0.28$; $t(779) = -1.20$, $p = 0.230$; SUDs: $ET_{Baseline} = -1.87$; $ET_{Follow\text{-}up} = -2.00$; $t(911) = 0.37$, p=0.713). This led to a stronger negative relationship between RTs and runway position at follow-up in SUDs compared to DEP/ANX at follow-up ($t(1200) = 2.59$, p=0.010), which was not present at baseline ($t(1539) = 0.37$, p=0.715).

Group differences in the relationship between response times and model parameters

LMEs were performed to examine potential group differences in the relationship between RTs and model parameters in the combined exploratory and confirmatory samples: *model parameter ~ group + time + RTs + group*time + group*RTs + group*time*RTs*. There was a reduction in *DU* over time ($F(1, 768.8) = 79.25$, $p < 0.001$; $EMM_{Baseline}$=1.08; $EMM_{Follow\text{-}up}$=0.78; $t(767) = 7.75$, $p < 0.001$), and a positive relationship between RTs and *DU* ($F(1, 1487.59) = 643.36$, $p < 0.001$) was found. As expected, a main effect of group ($F(2, 920.80)$=5.38, p=0.005) was found, whereby SUDs (EMM = 1.08) had greater *DU* than DEP/ANX (EMM = 0.94; $t(963) = -2.51$, $p = 0.012$) and HCs (EMM = 0.87; $t(927) = -2.93$, $p = 0.003$.

The LME was repeated for *EC*. There was also a reduction in *EC* over time ($F(1, 712.07) = 8.14$, $p = 0.005$; $EMM_{Baseline} = 0.74$; $EMM_{Folllow\text{-}up} = 0.81$; $t(709) = -2.49$, $p = 0.013$), and a positive relationship between RTs and *EC* ($F(1, 1555.99) = 83.77$, $p < 0.001$) was also present. There was a main effect of group ($F(2, 938.56) = 41.92$, $p < 0.001$), reflecting lower *EC* in the clinical sample (DEP/ANX: EMM = 0.82; $t(919) = 3.11$, $p = 0.002$; SUDs: EMM = 0.45; $t(949) = 8.16$, $p < 0.001$) when compared to HCs (EMM = 1.03). Within the clinical sample, SUDs had lower *EC* than DEP/ANX ($t(979) = 7.01$, $p < 0.001$). This LME also revealed a *group x RT* interaction ($F(2, 1554.12) = 3.48$, $p = 0.031$), suggesting a stronger positive association between RTs and *EC* in SUDs (ET = 0.69) than in HCs (ET = 0.17; $t(1556) = -2.64$, $p = 0.023$). Lastly, there was a *group x time* x *RT* interaction ($F(3, 812.34) = 3.41$, $p = 0.017$). This interaction was due to a decrease in the magnitude of the relationship between RTs and runway position over time in the DEP/ANX group ($ET_{Baseline} = 0.64$; $ET_{Follow\text{-}up} = 0.28$; $t(793) = -2.89$, $p = 0.004$), while the relationship between RTs and runway position remained relatively stable over time in the other two groups (HC: $ET_{Baseline} = 0.33$; $ET_{Follow\text{-}up} = 0.01$; $t(764) = 1.38$, $p = 0.17$; SUDs: $ET_{Baseline} = 0.69$; $ET_{Follow\text{-}up} = 0.70$; $t(887) = -0.04$, p=0.97). This led to a stronger positive relationship

between RTs and *EC* at follow-up in SUDs compared to DEP/ANX at follow-up ($t(1167) = -2.20$, $p=0.028$), which was not present at baseline ($t(1531) = -0.31$, $p=0.75$).

Supplementary Figures

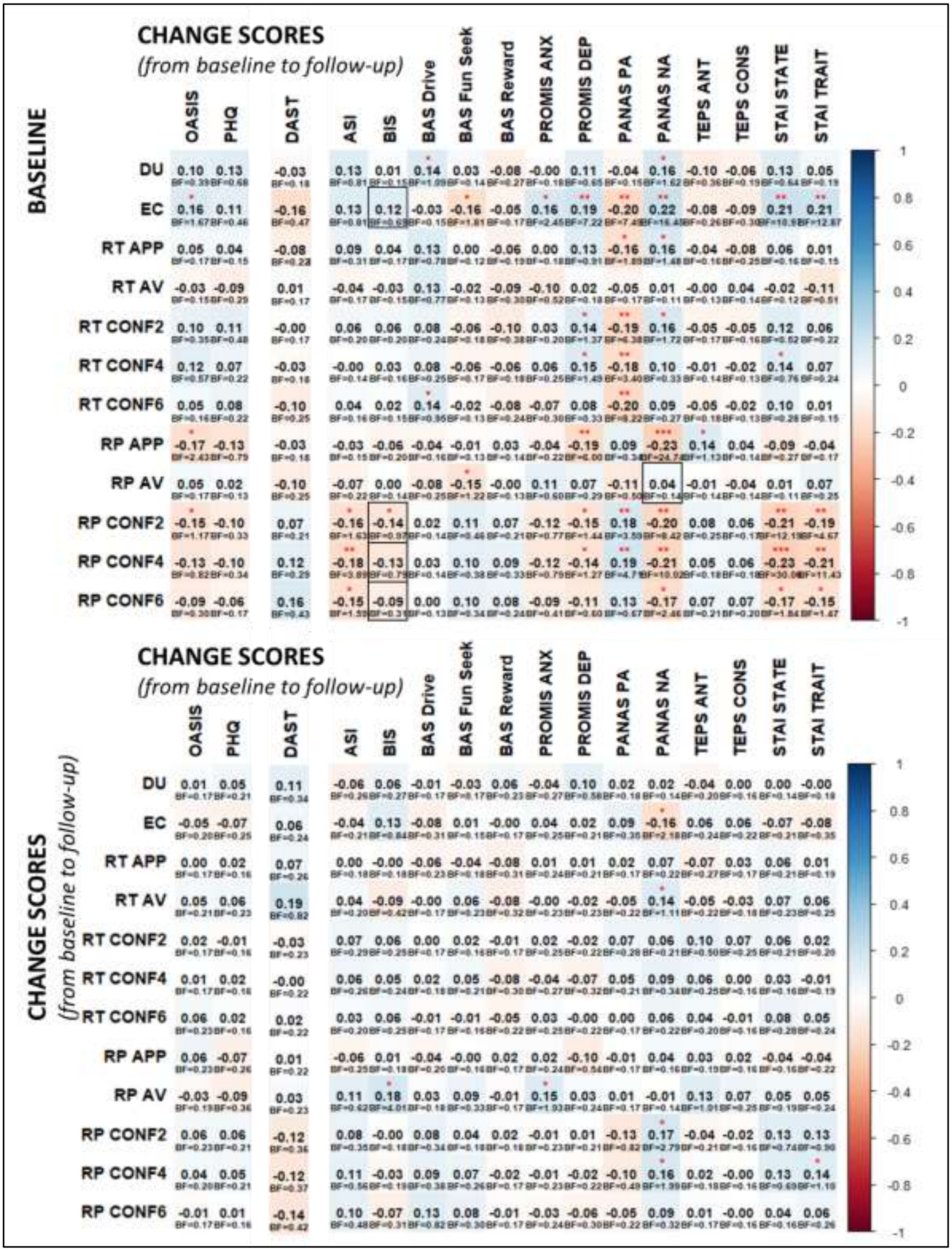

Supplementary Figure 1. (*Top*) Partial correlations between task measures at baseline and symptom change from baseline to follow-up, accounting for baseline symptom severity. (*Bottom*) Partial

correlations between changes over time in task measures and symptom severity, accounting for baseline symptom severity and baseline task performance *Left*: Relationships with clinical symptoms. *Right*: Relationships with dimensional measures as visualized in the *exploratory* paper. RT=response time; RP=average chosen runway position. Boxes represent significant partial correlations in the exploratory dataset. Uncorrected p-values: *$p < 0.001$, **$p < 0.01$, *$p < 0.05$. **Note:** For analyses involving DAST scores, data was included only for individuals who met the criteria for substance use disorders.

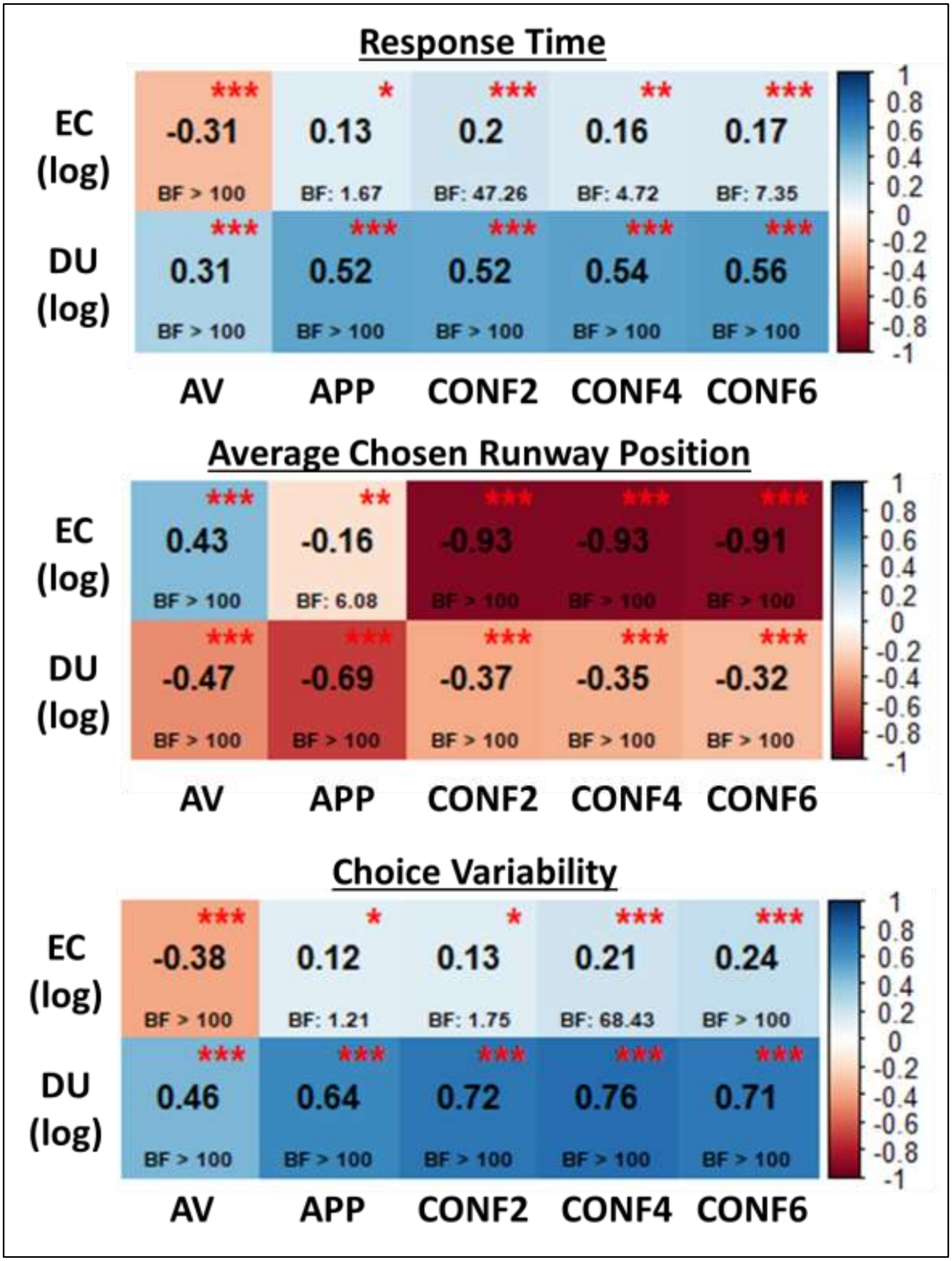

Supplementary Figure 2. Pearson correlations and associated Bayes factor values between model parameters and descriptive task measures (*top*: reaction time, *middle*: average chosen runway position, and *bottom*: choice variability). Uncorrected p-values: ***$p < 0.001$, **$p < 0.01$, *$p < 0.05$.

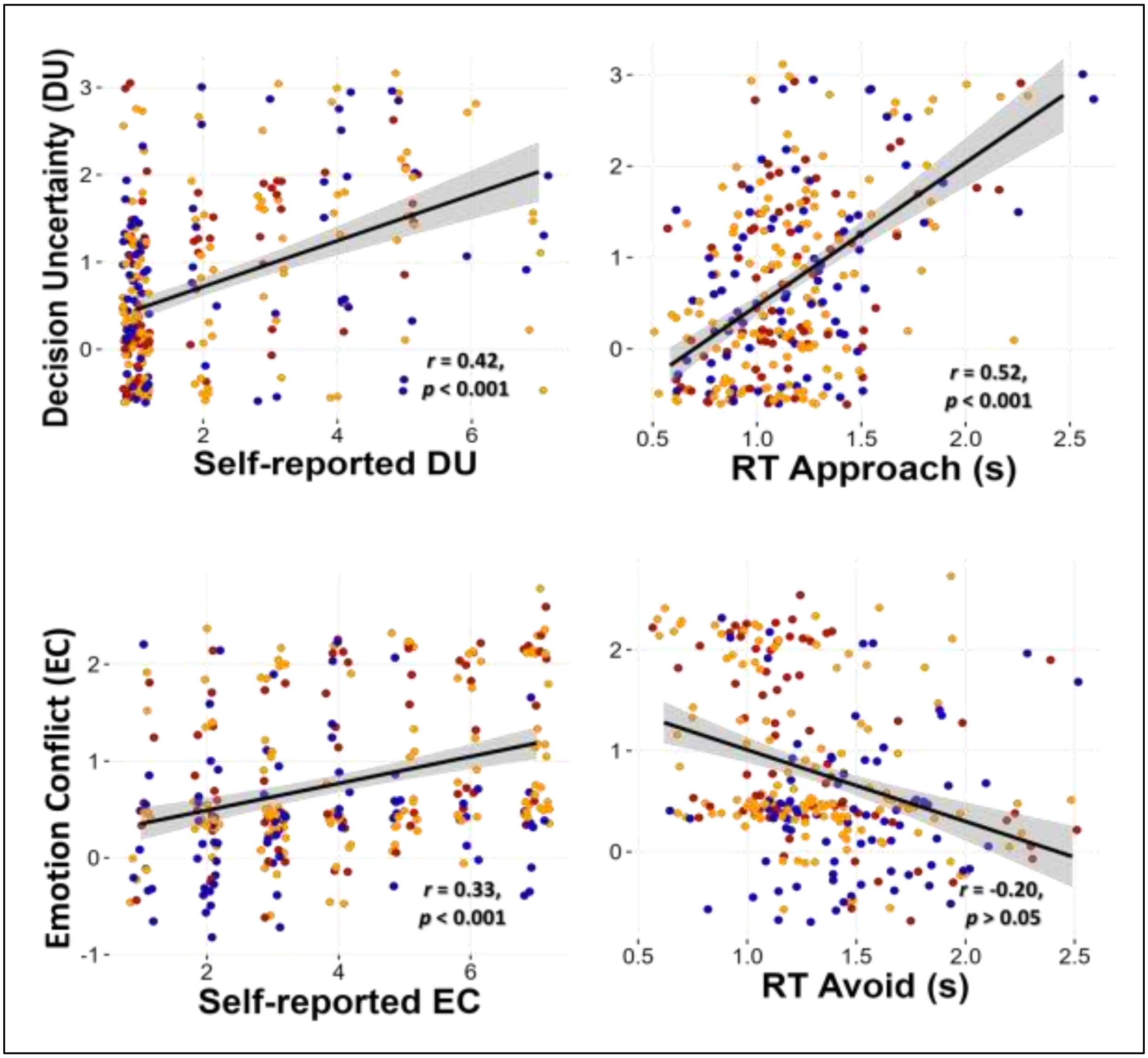

Supplementary Figure 3. Association between model parameters (*top*: *DU* and *bottom*: *EC*) and (*left*) select self-report questionnaire items (Q2 and Q3 in **Table 5**, main text) and (*right*) response time (RTs) in approach (*top*) and avoid (*bottom*) conditions.

Model Parameters	EC	DU
Group	**HCs > D/A > SUDs**	Exploratory:<br>SUDs > HCs; SUDs > D/A<br>**Confirmatory:**<br>**SUDs > D/A**
Time	T1 > T2	T1 > T2
Sex	F > M*	
Group x Sex	**Driven by females**	

Replication | Partial Replication | Not Replicated | New Findings | **Long-term Stability**

Supplementary Figure 4. Summary of replication and long-term stability of computational model parameters. Color coded boxes indicate replication status. Gray boxes indicate where effects were not found in the exploratory or confirmatory sample. Replication = congruent results in the exploratory and confirmatory samples. Partial Replication = replication of main effects or interactions but where not all post-hoc contrasts were replicated. Not Replicated = statistically significant results in the exploratory sample that were not significant in the confirmatory sample. New Findings = statistically significant results in the confirmatory sample that were not significant in the exploratory sample. Long-term Stability = bolding indicates statistically significant results at baseline (including all participants) that were also significant at follow-up in the exploratory sample. HCs = Healthy Comparisons; D/A = Depression and Anxiety; SUDs = Substance use disorder; T1 = Baseline (including all participants); T2 = Follow-up; F = Females; M = Males. *Indicates replication of main effects only. Specific post-hoc contrasts are from the present study.

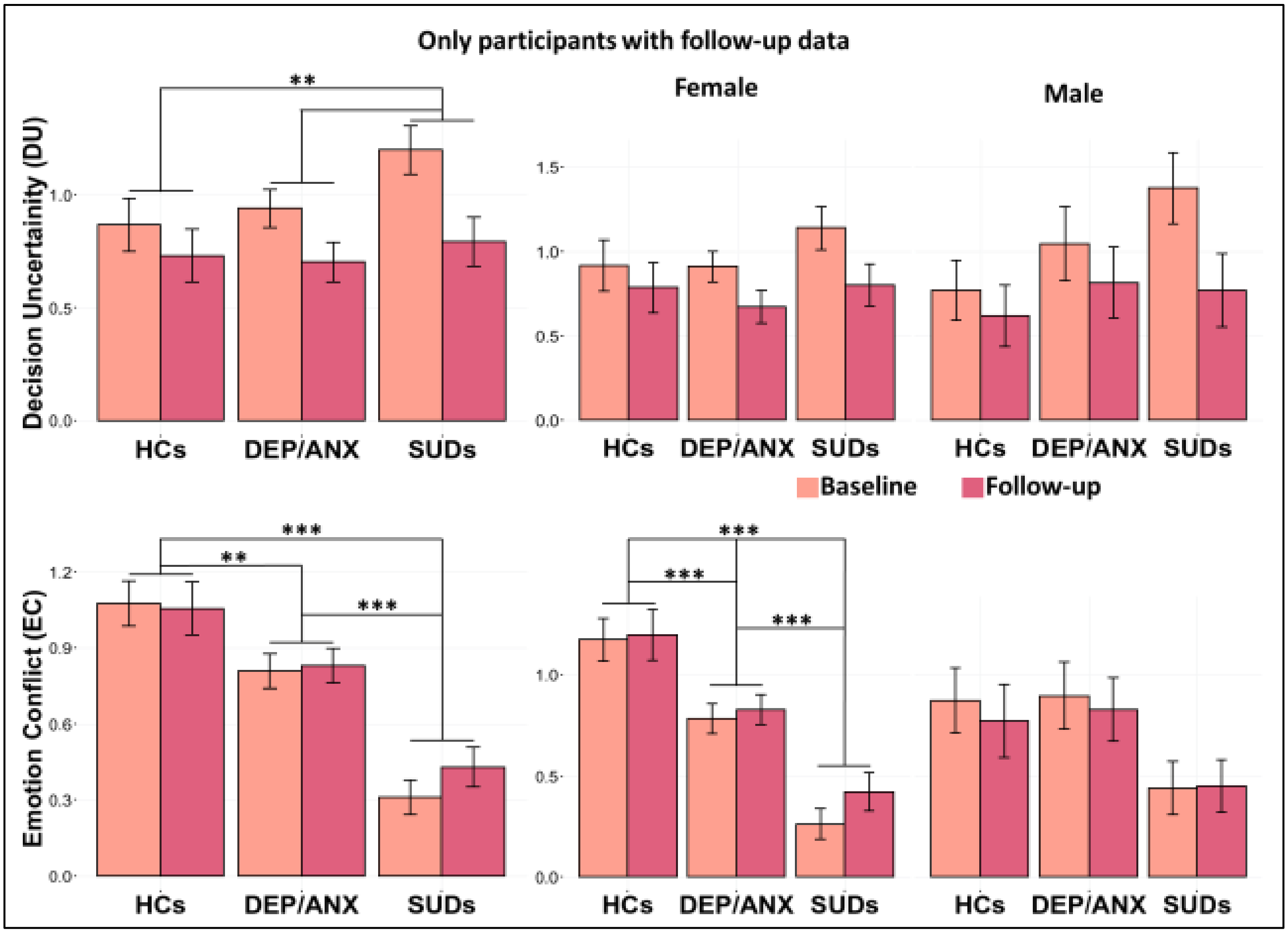

Supplementary Figure 5. Group differences are here visualized as bar plots (indicating means and standard errors) for only those participants who returned for follow-up. *Top*: All participants display lower decision uncertainty at follow-up. *Bottom*: SUDs display significantly lower emotion conflict; and the observed group differences appear to be driven by females. ***$p < 0.001$, **$p < 0.01$, *$p < 0.05$.

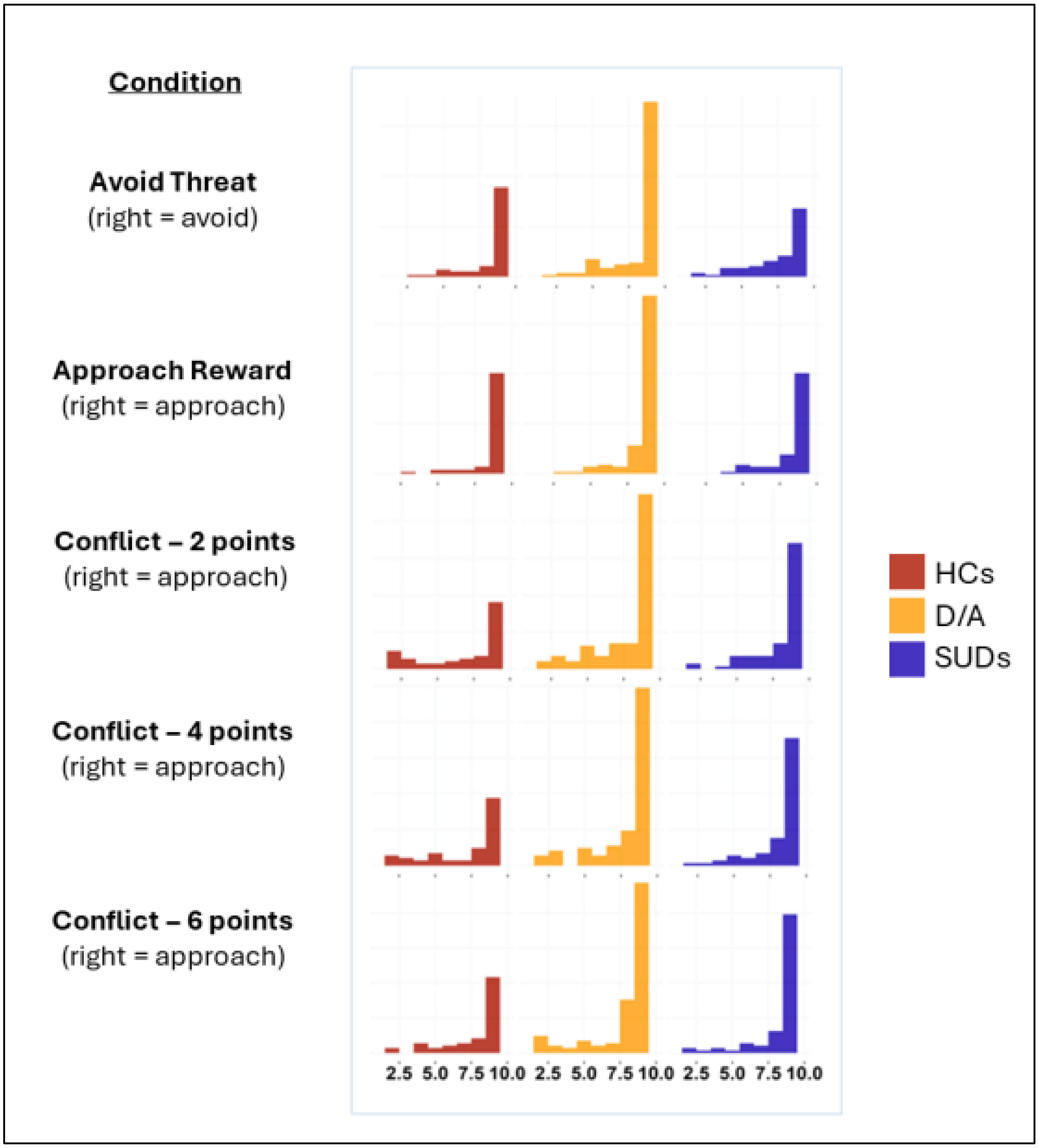

Supplementary Figure 6. Histograms depicting average chosen runway position separated by group and condition. The x-axis represents the frequency of chosen runway positions. In all but *avoid* trials, the right most position (position 10) indicates maximum approach behavior, while the left-most position (position 2) indicates maximum avoidance (*note*: position 1 was coded as the "undecided" position and is therefore not included here). HCs appeared to prefer either maximum approach or maximum avoidance depending on the trial type. In conflict trials, there appeared to be a moderate preference for avoidance that decreased with increasing point values. For D/As, as the conflict gradient increased, participants appeared to be more confident in their approach/avoidance preferences. Lower avoidance was observed for SUDs across all conditions relative to the other two groups.

RESPONSE TIME

Trial Type	ALL	APP	AV	CONF2	CONF4	CONF6
Group	SUDs > HCs<br>D/A > HCs	Exploratory:<br>SUDs > HCs; D/A > HCs<br>Confirmatory:<br>SUDs > D/A	Exploratory:<br>SUDs > HCs; D/A > HCs<br>**Confirmatory:**<br>**SUDs > HCs; SUDs > D/A**			
Time	T1 > T2	T1 > T2	T1 > T2	T1 > T2	T1 > T2	T1 > T2
Sex			F > M			
Group x Sex			**Females:**<br>**SUDs > D/A > HCs**<br>**Males:**<br>**SUDs > D/A; HCs > D/A**			

CHOSEN RUNWAY POSITION

Trial Type	ALL	APP	AV	CONF2	CONF4	CONF6
Group	HCs > SUDs; D/A > SUDs	HCs > SUDs<br>D/A > SUDs	Exploratory:<br>HCs > D/A > SUDs<br>**Confirmatory:**<br>**HCs > SUDs; D/A > SUDs**	Exploratory:<br>SUDs > HCs; D/A > HCs<br>**Confirmatory**<br>**SUDs > D/A > HCs**	Exploratory:<br>SUDs > HCs; D/A > HCs<br>**Confirmatory:**<br>**SUDs > D/A; SUDs > HCs**	Exploratory:<br>SUDs > HCs; D/A > HCs<br>**Confirmatory:**<br>**SUDs > D/A; SUDs > HCs**
Time		T2 > T1	T2 > T1			
Sex	M > F			Contrast only marginally significant M > F	Contrast only marginally significant M > F	Contrast only marginally significant M > F
Group x Sex				**Female:**<br>**SUDs > D/A > HCs**<br>**Males: NS**	**Female:**<br>**SUDs > D/A > HCs**<br>**Males: NS**	

CHOICE VARIABILITY

Trial Type	ALL	APP	AV	CONF2	CONF4	CONF6
Group		**SUDs > D/A; SUDs > HCs**	Exploratory:<br>SUDs > D/A > HCs<br>**Confirmatory:**<br>**SUDs > D/A; SUDs > HCs**	Exploratory:<br>SUDs > HCs; SUDs > D/A<br>**Confirmatory: NS**	Exploratory:<br>SUDs > HCs; SUDs > D/A<br>Confirmatory:<br>post-hocs NS	Exploratory:<br>SUDs > HCs; SUDs > D/A<br>Confirmatory: NS
Time	T1 > T2	T1 > T2	T1 > T2	T1 > T2	T1 > T2	T1 > T2
Sex	**F > M***					
Group x Sex						

Replication | Partial Replication | Not Replicated | New Findings | **Long-term Stability**

Supplementary Figure 7. Summary of replication and long-term stability of descriptive task measures by trial type. Color coded boxes indicate replication status. Gray boxes indicate where effects were not found in the exploratory or confirmatory sample. Replication = congruent results in the exploratory and confirmatory samples. Partial Replication = replication of main effects or interactions but where not all post-hoc contrasts were replicated. Not Replicated = statistically significant results in the exploratory sample that were not significant in the confirmatory sample. New Findings = statistically significant results in the confirmatory sample that were not significant in the exploratory sample. Long-term Stability = bolding indicates statistically significant results at baseline (including all participants) that were also significant at follow-up in the exploratory sample. HCs = Healthy Comparisons; D/A = Depression and Anxiety; SUDs = Substance use disorder; T1 = Baseline (including all participants); T2 = Follow-up; F = Females; M = Males. *Indicates significance found for main effects only.

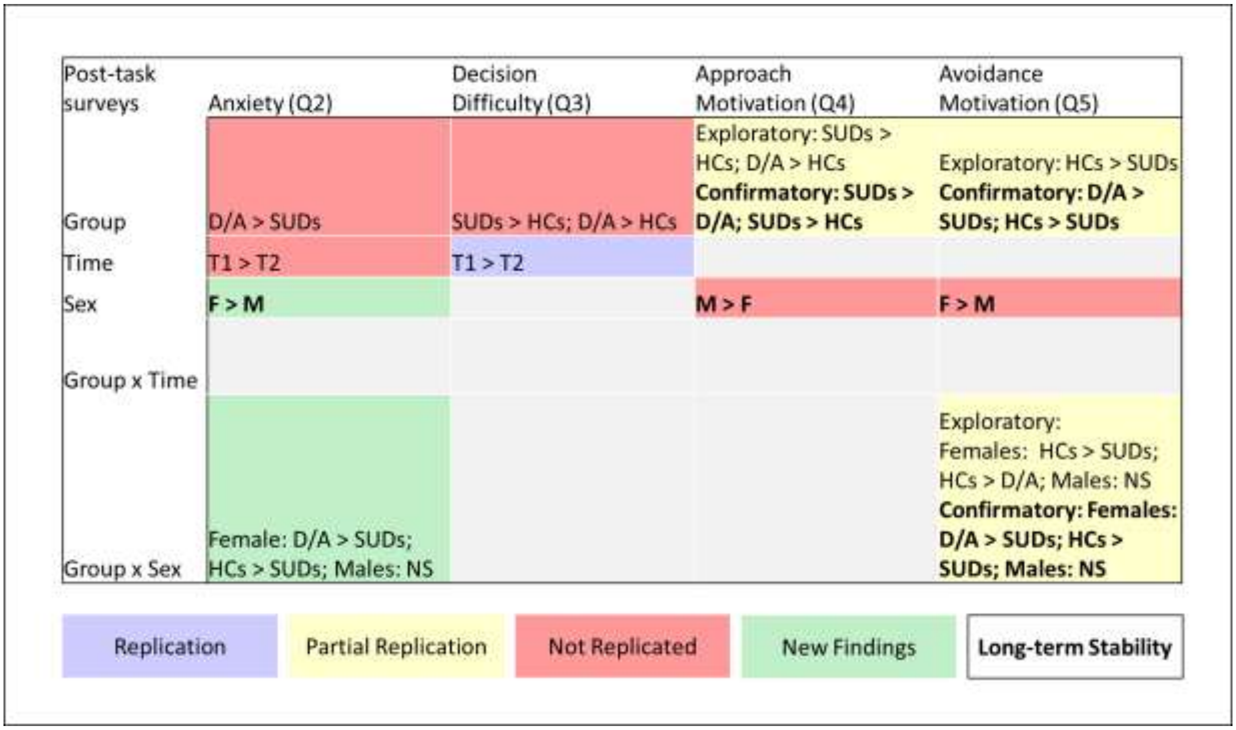

Post-task surveys	Anxiety (Q2)	Decision Difficulty (Q3)	Approach Motivation (Q4)	Avoidance Motivation (Q5)
Group	D/A > SUDs	SUDs > HCs; D/A > HCs	Exploratory: SUDs > HCs; D/A > HCs **Confirmatory: SUDs > D/A; SUDs > HCs**	Exploratory: HCs > SUDs **Confirmatory: D/A > SUDs; HCs > SUDs**
Time	T1 > T2	T1 > T2		
Sex	**F > M**		**M > F**	**F > M**
Group x Time				
Group x Sex	Female: D/A > SUDs; HCs > SUDs; Males: NS			Exploratory: Females: HCs > SUDs; HCs > D/A; Males: NS **Confirmatory: Females: D/A > SUDs; HCs > SUDs; Males: NS**

Supplementary Figure 8. **Summary of replication and long-term stability of self-reported post-task questionnaire items**. Color coded boxes indicate replication status. Gray boxes indicate where effects were not found in the exploratory or confirmatory sample. Replication = congruent results in the exploratory and confirmatory samples. Partial Replication = replication of main effects or interactions but where not all post-hoc contrasts were replicated. Not Replicated = statistically significant results in the exploratory sample that were not significant in the confirmatory sample. New Findings = statistically significant results in the confirmatory sample that were not significant in the exploratory sample. Long-term Stability = bolding indicates statistically significant results at baseline (including all participants) that were also significant at follow-up in the exploratory sample. HCs = Healthy Comparisons; D/A = Depression and Anxiety; SUDs = Substance use disorder; T1 = Baseline (including all participants); T2 = Follow-up; F = Females; M = Males.

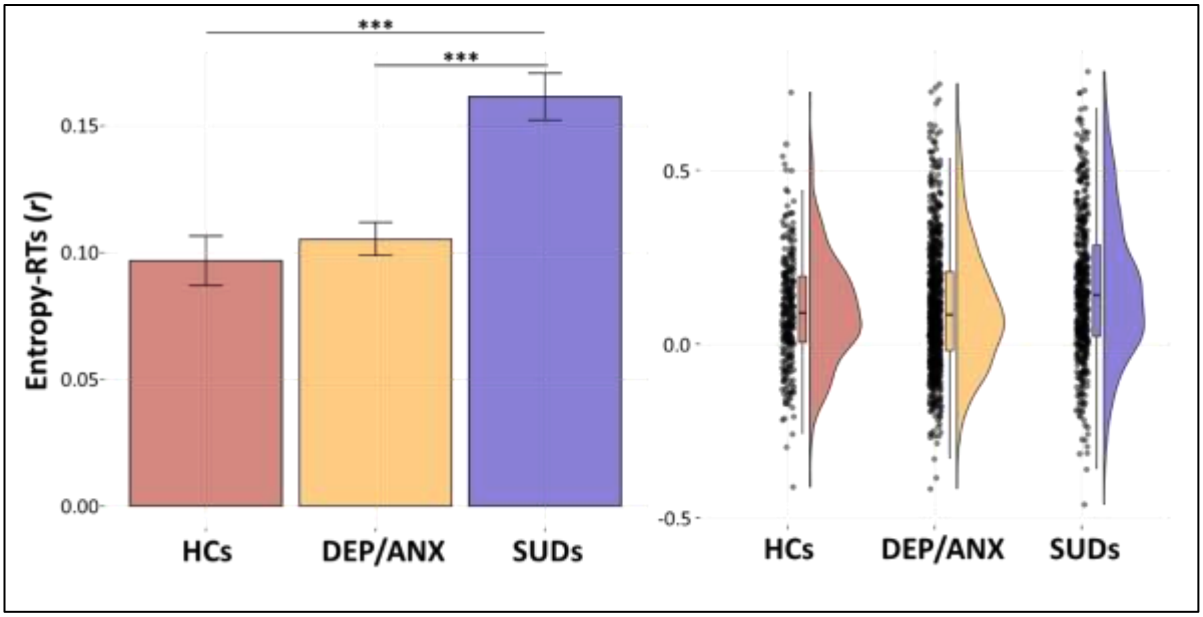

Supplementary Figure 9. Trial-by-trial measure of choice uncertainty (entropy) predicts response time. HCs = Healthy Comparisons; DEP/ANX = Depression and Anxiety; SUDs = Substance Use Disorder; ****p* < 0.001, ***p* < 0.01, **p* < 0.05.

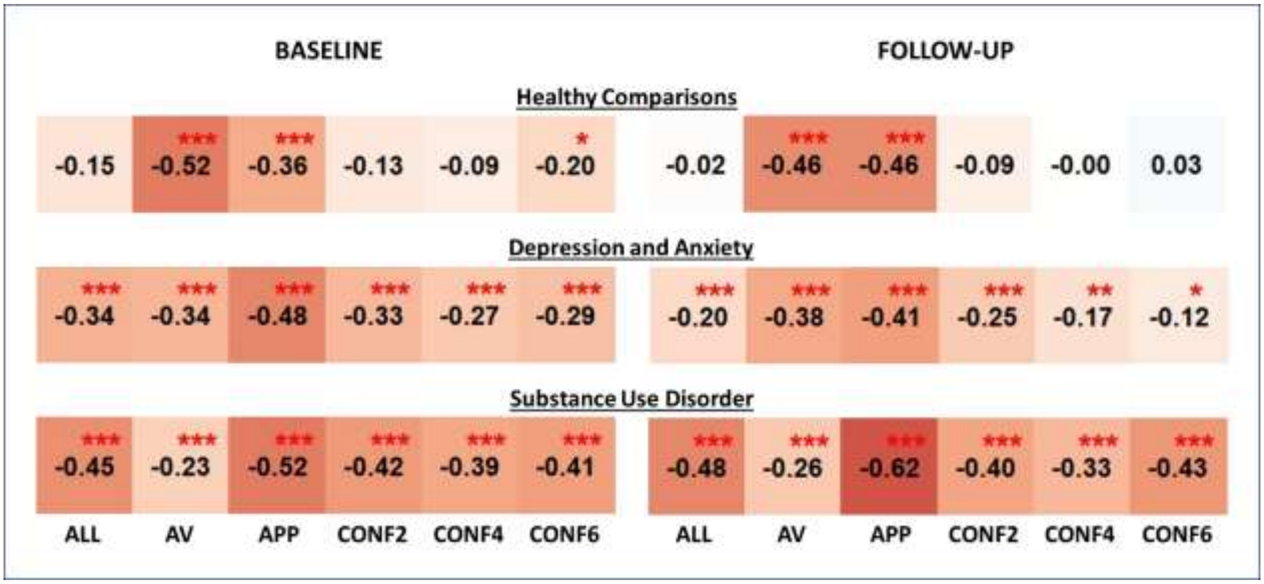

Supplementary Figure 10 Correlations between response time and chosen runway position were performed at baseline (*left*) and follow-up (*right*) for Healthy Comparisons (*top*), Depression and Anxiety (*middle*), and Substance Use Disorder (*bottom*) groups. Each cell represents a correlation between response time and chosen runway position either across all trials (ALL) or for a specific trial type (AV: avoid trials; APP: approach trials; CONF2: Conflict 2 trials; CONF4: Conflict 4 trials; CONF6: Conflict 6 trials). Un-corrected p-values: ****p* < 0.001, ***p* < 0.01, **p* < 0.05.

Supplementary Tables

Supplementary Table 1. Comorbidity table for participants who returned for follow-up

	MDD	GAD	SAD	PD	PTSD	Alc.	Can.	Stim.	Op.	Sed.	Halluc.
**MDD**	179										
**GAD**	78	91									
**SAD**	50	31	58								
**PD**	25	21	10	28							
**PTSD**	26	13	11	2	28						
**Alc.**	17	2	8	1	5	26					
**Can.**	17	8	10	0	2	11	29				
**Stim.**	47	16	16	3	7	24	25	72			
**Op.**	24	6	6	1	4	10	11	22	30		
**Sed.**	11	3	3	0	3	5	9	11	13	16	
**Halluc.**	1		0	0	0	1	0	1	0	0	1

Note: This table presents the composition of comorbidities across the clinical groups within the confirmatory sample. All data shown are restricted to participants who returned for follow-up. The composition for the full exploratory and confirmatory sample at baseline has been previously provided [1]. The diagonal contains the total number of participants with a given diagnosis (regardless of comorbidities). MDD: Major Depressive Disorder; GAD: Generalized Anxiety Disorder; SAD: Social Anxiety Disorder; PD: Panic Disorder; PTSD: Post-Traumatic Stress Disorder; Alc.: Alcohol; Can.: Cannabis; Stim.: Stimulant; Op.: Opioid; Sed.: Sedative; Halluc: Hallucinogens.

Supplementary Table 2. Differences in participant characteristics at baseline for participants who did vs. did not return for follow-up

	**Returned (N = 287)**	**Did Not Return (N = 193)**	***p***
Age	33.50 (9.96)	33.28 (9.53)	0.806
Sex (Male)	74 (25.80)	83 (43)	<0.001***
PHQ	8.14 (6.73)	7.99 (6.20)	0.801
OASIS	6.49 (4.89)	6.52 (4.85)	0.950
DAST-10	2.35 (3.44)	3.81 (4.01)	<0.001***
WRAT reading score	61.41 (6.40)	60.05 (6.14)	0.036*

Note: PHQ = Patient Health Questionnaire; OASIS = Overall Anxiety Severity and Impairment Scale; DAST = Drug Abuse Screening Test
p*-values are based on t-tests for significant differences between the three groups (**p* < 0.001; ***p* < 0.01; **p* < 0.05). Sex is reported in terms of counts and percentages. *p*-values are based on associated Chi-Squared statistics.

Supplementary Table 3. Baseline symptom and demographic characteristics by group in participants who did vs. did not return for follow-up

	**HCs**		**DEP/ANX**		**SUDs**	
	**Returned**	**Did Not Return**	**Returned**	**Did Not Return**	**Returned**	**Did Not Return**
N	69	28	135	73	83	92
Age	33.45	28.75	33.63 (10.13)	33.94	33.34	34.13 (8.37)

	(11.29)	(10.06)		(10.32)	(8.52)	
Sex (Male)	23 (33.3)	15 (53.6)	29 (21.5)	22 (30.1)	22 (26.5)+	46 (50.0)+
PHQ	1.07 (1.75)	1.61 (1.97)	12.74 (5.08)	12.14 (4.86)	6.54 (5.81)	6.64 (5.75)
OASIS	0.96 (1.49)	1.46 (1.88)	9.77 (3.46)	9.42 (3.82)	5.77 (4.24)	5.76 (4.67)
DAST	0.19 (0.52)	0.18 (0.39)	0.44 (0.98)	0.37 (0.89)	7.25 (2.28)	7.65 (2.18)
WRAT reading score	63.25 (5.63)	61.76 (4.38)	63.02 (5.03)	62.31 (5.11)	57.12 (7.12)	56.38 (6.36)

Note: PHQ = Patient Health Questionnaire; OASIS = Overall Anxiety Severity and Impairment Scale; DAST = Drug Abuse Screening Test; +Sex is reported in terms of counts and percentages. *p*-values are based on associated Chi-Squared statistics.

Supplementary Table 4. Group-wise differences in participant characteristics at 1-year follow-up in the exploratory and confirmatory sample

	**HCs**		**DEP/ANX**		**SUDs**	
	Exploratory	***Confirmatory***	***Exploratory***	***Confirmatory***	***Exploratory***	***Confirmatory***
N	48	69	192	135	84	83
Age	32.94 (11.29)	34.50 (11.30)	37.15 (11.40)*	34.69 (10.17)*	36.28 (9.18)	34.41 (8.56)
Sex (Male)	24 (50.0)	23 (33.3)	50 (26.0)	29 (21.5)	34 (40.5)	22 (26.5)
PHQ	1.14 (1.84)	0.91 (1.73)	8.27 (6.10)	8.47 (6.10)	3.10 (4.57)	3.30 (4.44)
OASIS	1.47 (2.31)	0.86 (1.75)	7.56 (4.58)	7.11 (4.)	3.54 (4.36)	3.26 (3.73)
DAST	0.47 (0.56)	0.30 (0.55)	0.56 (1.05)	0.37 (0.6)	2.46 (2.89)	2.27 (2.53)
WRAT reading score	63.89 (4.54)	63.25 (5.63)	63.03 (4.64)	63.02 (5.03)	58.49 (6.00)	57.12 (7.12)

Note: PHQ = Patient Health Questionnaire; OASIS = Overall Anxiety Severity and Impairment Scale; DAST = Drug Abuse Screening Test; **p* < 0.05 based on t-test for significant differences between samples within each group. + Sex is reported in terms of counts and percentages. *p*-values are based on associated Chi-Squared statistics.

Supplementary Table 5. Group-wise intra-class correlations for clinical measures at baseline and 1-year follow-up

**Group**	**Measure**	**ICC value**	**F-value**	**df1, df2**
**All**	PHQ	0.64***	4.567	281,281
	OASIS	0.58***	3.802	281,281
	DAST	0.42***	2.468	281,281
	ASI	0.67***	4.989	279,279
	BIS (Inhibition)	0.74***	6.613	279,279
	BAS (Drive)	0.60***	4.054	279,279
	BAS (Fun Seeking)	0.62***	4.302	279,279
	BAS (Reward)	0.53***	3.213	279,279
	PROMIS (Anxiety)	0.64***	4.529	280,280

	PROMIS (Depression)	0.63***	4.456	281,281
	PANASX (PA)	0.56***	3.568	280,280
	PANASX (NA)	0.61***	4.167	280,280
	TEPS (Anticipatory)	0.61***	4.130	280,280
	TEPS (Consummatory)	0.70***	5.671	280,280
	STAI (State)	0.60***	3.999	278,278
	STAI (Trait)	0.72***	6.085	278,278
**HCs**	PHQ	0.16	1.387	68,68
	OASIS	0.13	1.287	68,68
	DAST	0.36***	2.124	68,68
	ASI	0.55***	3.477	68,68
	BIS (Inhibition)	0.75***	7.096	68,68
	BAS (Drive)	0.46***	2.702	68,68
	BAS (Fun Seeking)	0.66***	4.925	68,68
	BAS (Reward)	0.51***	3.077	68,68
	PROMIS (Anxiety)	0.49***	2.912	68,68
	PROMIS (Depression)	0.39***	2.289	68,68
	PANASX (PA)	0.60***	4.003	68,68
	PANASX (NA)	0.52***	3.139	68,68
	TEPS (Anticipatory)	0.61***	4.121	68,68
	TEPS (Consummatory)	0.71***	5.986	68,68
	STAI (State)	0.70***	5.692	68,68
	STAI (Trait)	0.84***	11.566	68,68
**DEP/ANX**	PHQ	0.42***	2.442	130,130
	OASIS	0.39***	2.267	130,130
	DAST	0.29***	1.813	130,130
	ASI	0.58***	3.781	128,128
	BIS (Inhibition)	0.70***	5.576	128,128
	BAS (Drive)	0.68***	5.332	128,128
	BAS (Fun Seeking)	0.68***	5.206	128,128
	BAS (Reward)	0.60***	4.010	128,128
	PROMIS (Anxiety)	0.40***	2.353	129,129
	PROMIS (Depression)	0.44***	2.575	130,130
	PANASX (PA)	0.48***	2.813	129,129
	PANASX (NA)	0.49***	2.954	129,129
	TEPS (Anticipatory)	0.74***	6.599	129,129
	TEPS (Consummatory)	0.73***	6.348	129,129
	STAI (State)	0.45***	2.645	127,127
	STAI (Trait)	0.49***	2.906	127,127
**SUDs**	PHQ	0.45***	2.666	81,81
	OASIS	0.19*	1.481	81,81
	DAST	0.16	1.384	81,81
	ASI	0.52***	3.143	81,81
	BIS (Inhibition)	0.56***	3.512	81,81
	BAS (Drive)	0.57***	3.633	81,81
	BAS (Fun Seeking)	0.52***	3.150	81,81
	BAS (Reward)	0.40***	2.323	81,81
	PROMIS (Anxiety)	0.43***	2.539	81,81
	PROMIS (Depression)	0.37***	2.178	81,81

PANASX (PA)	0.32**	1.953	81,81
PANASX (NA)	0.54***	3.298	81,81
TEPS (Anticipatory)	0.37***	2.188	81,81
TEPS (Consummatory)	0.65***	4.640	81,81
STAI (State)	0.37***	2.164	81,81
STAI (Trait)	0.50***	3.015	81,81

Note: PHQ = Patient Health Questionnaire; OASIS = Overall Anxiety Severity and Impairment Scale; DAST = Drug Abuse Screening Test; ASI = Anxiety Sensitivity Index; BIS/BAS = Behavioral Activation/Inhibition scale with subscales on Inhibition (BIS), Drive (BAS), Fun Seeking (BAS) and Reward (BAS); PROMIS = Patient-Reported Outcomes Measurement Information System with depression and anxiety subscales; PANAS = Positive and Negative Affect Schedule: Positive Affect (PA) and Negative Affect (NA); TEPS = Temporal Experience of Pleasure Scale (Anticipatory and Consummatory subscales); STAI = State-Trait Anxiety Inventory (subscales for State and Trait anxiety). ***$p < 0.001$, **$p < 0.01$, *$p < 0.05$.

Supplementary Table 6. Group-wise intra-class correlations for post-task questionnaire items at baseline and 1-year follow-up

**Post-Task Self-Report Questions (Likert Scale: 1 = not at all; 7 = very much)**	**All** (Baseline=480; Follow-up=287)	**HCs** (Baseline=97; Follow-up=69)	**DEP/ANX** (Baseline=208; Follow-up=135)	**SUDs** (Baseline=175; Follow-up=83)
Q1. I found the positive pictures enjoyable	ICC=0.47; $F(286,286)=2.80$, $p<0.001$***	ICC=0.53; $F(68,68)=3.25$, $p<0.001$***	ICC=0.47; $F(134,134)=2.80$, $p<0.001$***	ICC=0.42; $F(82,82)=2.47$, $p<0.001$***
Q2. The negative pictures made me feel anxious or uncomfortable	ICC=0.52; $F(286,286)=3.15$, $p<0.001$***	ICC=0.47; $F(68,68)=2.77$, $p<0.001$***	ICC =0.52; $F(134,134)=3.20$, $p<0.001$***	ICC=0.53; $F(82,82)=3.27$, $p<0.001$***
Q3. I often found it difficult to decide which outcome I wanted	ICC=0.40; $F(286,286)=2.33$, $p<0.001$***	ICC=0.33; $F(68,68)=1.99$, $p=0.003$**	ICC=0.45; $F(134,134)=2.63$, $p<0.001$***	ICC=0.36; $F(82,82)=2.13$, $p<0.001$***
Q4. I always tried to move all the way towards the outcome with the largest reward points	ICC=0.59; $F(286,286)=3.90$, $p<0.001$***	ICC=0.68; $F(68,68)=5.33$, $p<0.001$***	ICC=0.68; $F(134,134)=5.18$, $p<0.001$***	ICC=0.17; $F(82,82)=1.40$, $p=0.06$
Q5. I always tried to move all the way away from the outcome with the negative picture/sounds	ICC=0.52; $F(286,286)=3.21$, $p<0.001$***	ICC=0.57; $F(68,68)=3.70$, $p<0.001$***	ICC=0.65; $F(134,134)=4.66$, $p<0.001$***	ICC=0.12; $F(82,82)=1.26$, $p=0.15$
Q6. When a negative picture and sound were	ICC=0.55; $F(286,286)=3.40$, $p<0.001$***	ICC=0.64; $F(68,68)=4.51$, $p<0.001$***	ICC=0.59; $F(134,134)=3.89$, $p<0.001$***	ICC=0.31; $F(82,82)=1.91$, $p=0.002$**

displayed, I kept my eyes open and looked at the picture				
Q7. When a negative picture and sound were displayed, I tried to think about something unrelated to the picture to distract myself	ICC =0.50; *F*(286,286)=2.97, *p*<0.001***	ICC=0.53; *F*(68,68)=3.26, *p*<0.001***	ICC=0.51; *F*(134,134)=3.05, *p*<0.001***	ICC=0.36; *F*(82,82)=2.11, *p*<0.001***
Q8. When a negative picture and sound were displayed, I tried other strategies to manage emotions triggered by the pictures	ICC=0.55; *F*(286,286)=3.46, *p*<0.001***	ICC=0.61; *F*(68,68)=4.12, *p*<0.001***	ICC=0.54; *F*(134,134)=3.33, *p*<0.001***	ICC=0.46; *F*(82,82)=2.72, *p*<0.001***

Note: ***$p < 0.001$, **$p < 0.01$, *$p < 0.05$.

Supplementary Table 7. Group-wise post-task self-report questionnaire items at baseline and follow-up, and correlations with computational model parameters at follow-up

Post-Task Self-Report Questions (Likert Scale: 1 = not at all; 7 = very much)	Mean (SD) Baseline		Mean (SD) 1-Year follow-up	Emotion conflict (EC)	Decision uncertainty (β)
	All participants	Returned for follow-up			
**HCs (N_{All}= 97; $N_{Follow-up}$=69)**					
Q1. I found the positive pictures enjoyable	5.31 (1.39)	5.51 (1.30)	5.20 (1.62)	0.23	-0.01
Q2. The negative pictures made me feel anxious or uncomfortable	4.01 (2.01)	3.99 (1.94)	4.04 (1.92)	0.47***	-0.21
Q3. I often found it difficult to decide which outcome I wanted	2.11 (1.66)	2.16 (1.63)	1.86 (1.29)	-0.05	0.51***
Q4. I always tried to move all the way towards the outcome with the largest reward points	4.34 (2.55)	4.10 (2.51)	4.38 (2.56)	-0.89***	-0.33**
Q5. I always tried to move all the way away from the outcome with the negative picture/sounds	3.24 (2.37)	3.36 (2.35)	3.30 (2.51)	0.75***	0.12
Q6. When a negative picture and sound were displayed, I kept my eyes open and looked at the picture	5.13 (2.01)	5.16 (1.96)	4.91 (2.09)	-0.42***	-0.08
Q7. When a negative picture and sound were displayed, I tried to think about something unrelated to the picture to distract myself	3.14 (2.07)	3.16 (2.06)	3.41 (2.00)	0.46***	0.10

Q8. When a negative picture and sound were displayed, I tried other strategies to manage emotions triggered by the pictures	3.28 (1.92)	3.45 (1.91)	3.62 (1.93)	0.46***	-0.05
DEP/ANX (N_{All}= 208; $N_{Follow-up}$=135)					
Q1. I found the positive pictures enjoyable	5.05 (1.53)	5.12 (1.56)	5.09 (1.51)	0.09	-0.13
Q2. The negative pictures made me feel anxious or uncomfortable	4.18 (1.96)	4.13 (2.03)	4.25 (1.94)	0.32***	0.04
Q3. I often found it difficult to decide which outcome I wanted	2.31 (1.71)	2.27 (1.70)	2.12 (1.67)	0.01	0.44***
Q4. I always tried to move all the way towards the outcome with the largest reward points	4.70 (2.40)	4.73 (2.43)	4.90 (2.52)	-0.83***	-0.48***
Q5. I always tried to move all the way away from the outcome with the negative picture/sounds	3.12 (2.25)	3.10 (2.27)	2.99 (2.35)	0.79***	0.33***
Q6. When a negative picture and sound were displayed, I kept my eyes open and looked at the picture	5.25 (1.92)	5.23 (2.00)	5.28 (1.97)	-0.43***	-0.29***
Q7. When a negative picture and sound were displayed, I tried to think about something unrelated to the picture to distract myself	3.15 (1.93)	3.13 (1.93)	3.14 (2.03)	0.32***	0.07
Q8. When a negative picture and sound were displayed, I tried other strategies to manage emotions triggered by the pictures	3.37 (1.94)	3.30 (1.91)	3.32 (1.93)	0.32***	-0.01
SUDs (N_{All}= 175; $N_{Follow-up}$=83)					
Q1. I found the positive pictures enjoyable	4.83 (1.66)	4.99 (1.71)	4.59 (1.64)	-0.14	-0.12
Q2. The negative pictures made me feel anxious or uncomfortable	3.80 (1.96)	3.73 (1.94)	3.54 (1.94)	0.16	-0.18
Q3. I often found it difficult to decide which outcome I wanted	2.45 (1.75)	2.34 (1.71)	2.17 (1.73)	0.02	0.36***
Q4. I always tried to move all the way towards the outcome with the largest reward points	5.36 (2.08)	5.63 (1.94)	5.63 (2.05)	-0.73***	-0.6***
Q5. I always tried to move all the way away from the outcome with the negative picture/sounds	2.33 (1.79)	2.27 (1.76)	2.51 (2.06)	0.53***	0.24*
Q6. When a negative picture and sound were displayed, I kept my eyes open and looked at the picture	5.82 (1.72)	5.86 (1.59)	5.61 (1.83)	-0.26*	-0.12
Q7. When a negative picture and sound were displayed, I tried to think about something unrelated to the picture to distract myself	2.29 (1.65)	2.41 (1.80)	2.27 (1.65)	0.2	0
Q8. When a negative picture and	2.51 (1.76)	2.53 (1.79)	2.71 (1.84)	0.09	-0.09

sound were displayed, I tried other strategies to manage emotions triggered by the pictures			

Note: ***$p < 0.001$, **$p < 0.01$, *$p < 0.05$.

Supplementary Table 8. Results of linear mixed effects models predicting *DU* and *EC* in data including all baseline participants, when accounting for effects of group and time

Predictor*	Result	EMM	Post-hoc contrast
		**Decision Uncertainty (*DU*)**	
Group	$F(2, 493)=1.44$, $p=0.239$		NS
Time	$F(1, 337)$ =24.16, $p<0.001$	T1=1.022; T2=0.745	T1 - T2: $t(340.3)=5.296$, $p<0.001$, $d=0.43$
Group x Time	$F(2, 337)=1.60$, $p=0.203$		NS
		**Emotion Conflict (*EC*)**	
Group	$F(2, 495)=20.64$, $p<0.001$	D/A=0.861; HCs=1.022; SUDs=0.465	D/A - HCs: $t(458.3)=-1.826$, $p=0.068$, $d=-0.36$<br>D/A - SUDs: $t(485.37)=5.28$, $p<0.001$, $d=0.9$<br>HCs - SUDs: $t(472.44)=6.072$, $p<0.001$, $d=1.26$
Time	$F(1, 325)=0.50$, $p=0.478$		NS
Group x Time	$F(2, 325)=0.48$, $p=0.620$		NS

Note: HCs = Healthy Comparisons; D/A = Depression and Anxiety; SUDs = Substance Use Disorder; T1 = Baseline; T2 = Follow-up; NS = nonsignificant.
For interpretability, sum coding was used for time (baseline = −1; follow-up = 1), and group (with HCs coded as -1).

Supplementary Table 9. Results of linear mixed effects models predicting *DU* and *EC* in data including all baseline participants, when accounting for effects of group, time, age, sex, and WRAT scores

Predictor*	Result	EMM	Post-hoc contrast
		**Decision Uncertainty (*DU*)**	
Group	$F(2, 376)=0.49$, $p=0.615$		NS
Time	$F(1, 289)=26.54$, $p<0.001$	T1=1.029; T2=0.732	T1 - T2: $t(289.57)=5.152$, $p<0.001$, $d=0.45$
Age	$F(1, 367)=21.13$, $p<0.001$		
Sex	$F(1, 383)=1.70$, $p=0.193$		NS
WRAT	$F(1, 361)=19.70$,		

	p<0.001		
Group x Age	*F*(2, 372)=1.63, *p*=0.197		NS
Group x Sex	*F*(2, 381)=1.32, *p*=0.267		NS
Group x WRAT	*F*(2, 364)=1.64, *p*=0.195		NS
**Emotion Conflict (*EC*)**			
Group	*F*(2, 378)=12.55, *p*<0.001	D/A=0.792; HCs=1.137; SUDs=0.344	D/A - HCs: *t*(364.38)=-3.474, *p*<0.001, *d*=-0.82<br>D/A - SUDs: *t*(379.98)=4.881, *p*<0.001, *d*=1.06<br>HCs - SUDs: *t*(366.6)=7.093, *p*<0.001, *d*=1.87
Time	*F*(1, 275)=0.28, *p*=0.598		NS
Age	*F*(1, 372)=4.88, *p*=0.028		
Sex	*F*(1, 383)=8.37, *p*=0.004	Female=0.789; Male=0.564	Female - Male: *t*(386.87)=2.632, *p*<0.01, *d*=0.53
WRAT	*F*(1, 367)=1.75, *p*=0.187		NS
Group x Age	*F*(2, 380)=0.41, *p*=0.664		NS
Group x Sex	*F*(2, 382)=4.67, *p*=0.010	Female:<br>D/A=0.848; HCs=1.283; SUDs=0.322;<br>Male<br>D/A=0.61; HCs=0.67; SUDs=0.414	Female:<br>D/A - HCs: *t*(361.18)=-3.733, *p*<0.001, *d*=-1.03<br>D/A - SUDs: *t*(377.07)=5.012, *p*<0.001, *d*=1.24<br>HCs - SUDs: *t*(364.45)=7.336, *p*<0.001, *d*=2.27<br>Male:<br>D/A - HCs: *t*(380.68) =-0.358, *p*=0.721, *d*=-0.14<br>D/A - SUDs: *t*(395.1)=1.065, *p*=0.288, *d*=0.46<br>HCs - SUDs: *t*(385.67)=1.301, *p*=0.194, *d*=0.6
Group x WRAT	*F*(2, 370)=5.56, *p*=0.004	D/A=0.035; HCs=0.003; SUDs=-0.011	D/A - HCs: *t*(365)=1.827, *p*=0.162<br>D/A - SUDs: *t*(381)=3.295, *p*=0.003, *d*=0.11<br>HCs - SUDs: *t*(362)=0.765,

p=0.725

Note: HCs = Healthy Comparisons; D/A = Depression and Anxiety; SUDs = Substance Use Disorder; T1 = Baseline; T2 = Follow-up; NS = nonsignificant. For interpretability, age and WRAT scores were centered; sum coding was used for sex (female = −1; male = 1), time (baseline = −1; follow-up = 1), and group (with HCs coded as -1).

Supplementary Table 10. Summary statistics for response times (Mean (SD)) at baseline and follow-up

**Full Sample**	**HCs (N = 97)**	**DEP/ANX (N = 208)**	**SUDs (N = 175)**	***p****
**All trials**				
Baseline	1.24 (0.27)	1.25 (0.29)	1.30 (0.33)	0.169
Follow-up	1.19 (0.30)	1.19 (0.29)	1.23 (0.32)	0.598
**Approach**				
Baseline	1.20 (0.30)	1.22 (0.35)	1.29 (0.40)	0.051
Follow-up	1.15 (0.33)	1.16 (0.33)	1.19 (0.34)	0.735
**Avoid**				
Baseline	1.30 (0.35)	1.35 (0.31)	1.51 (0.35)	<0.001***
Follow-up	1.29 (0.40)	1.30 (0.35)	1.45 (0.32)	0.007**
**CONF 2**				
Baseline	1.25 (0.31)	1.25 (0.34)	1.26 (0.38)	0.886
Follow-up	1.17 (0.33)	1.18 (0.32)	1.19 (0.38)	0.930
**CONF 4**				
Baseline	1.19 (0.31)	1.22 (0.37)	1.21 (0.36)	0.831
Follow-up	1.14 (0.29)	1.16 (0.33)	1.16 (0.37)	0.938
**CONF 6**				
Baseline	1.24 (0.30)	1.23 (0.33)	1.22 (0.37)	0.945
Follow-up	1.18 (0.32)	1.17 (0.33)	1.17 (0.36)	0.968
**Participants who Returned for Follow-up**	**HCs (N=69)**	**DEP/ANX (N=135)**	**SUDs (N=83)**	***p****
**All trials**				
Baseline	1.24 (0.27)	1.25 (0.28)	1.28 (0.35)	0.611
Follow-up	1.19 (0.30)	1.19 (0.29)	1.23 (0.32)	0.598
**Approach**				
Baseline	1.20 (0.30)	1.21 (0.33)	1.29 (0.45)	0.193
Follow-up	1.15 (0.32)	1.16 (0.33)	1.19 (0.34)	0.735
**Avoid**				
Baseline	1.27 (0.34)	1.37 (0.31)	1.51 (0.38)	<0.001***
Follow-up	1.29 (0.40)	1.30 (0.35)	1.45 (0.32)	0.007**
**CONF 2**				
Baseline	1.26 (0.33)	1.24 (0.34)	1.23 (0.37)	0.872
Follow-up	1.17 (0.33)	1.18 (0.32)	1.19 (0.38)	0.930
**CONF 4**				
Baseline	1.21 (0.34)	1.22 (0.37)	1.19 (0.37)	0.798
Follow-up	1.14 (0.29)	1.16 (0.33)	1.16 (0.37)	0.938
**CONF 6**				
Baseline	1.24 (0.30)	1.21 (0.29)	1.19(0.39)	0.679

Follow-up	1.18 (0.32)	1.17 (0.33)	1.17 (0.36)	0.968

Note: ****p* < 0.001, ***p* < 0.01, **p* < 0.05.

Supplementary Table 11. Summary statistics for average chosen runway positions (Mean (SD)) at baseline and follow-up

**Full Sample**	**HCs (N = 97)**	**DEP/ANX (N = 208)**	**SUDs (N = 175)**	***p****
**All trials**				
Baseline	6.98 (1.92)	7.20 (1.81)	7.51 (1.43)	0.039
Follow-up	6.85 (2.00)	7.38 (1.90)	7.73 (1.54)	0.013*
**Approach**				
Baseline	8.43 (1.33)	8.20 (1.63)	8.01 (1.55)	0.092*
Follow-up	8.57 (1.12)	8.45 (1.28)	8.31 (1.29)	0.421
**Avoid**				
Baseline	8.43 (1.33)	8.01 (1.62)	6.78 (2.07)	<0.001***
Follow-up	8.29 (1.44)	8.21 (1.53)	7.25 (2.28)	<0.001***
**CONF 2**				
Baseline	5.75 (3.12)	6.25 (2.94)	7.32 (2.14)	<0.001***
Follow-up	5.53 (3.41)	6.60 (3.06)	7.53 (2.38)	<0.001***
**CONF 4**				
Baseline	6.13 (3.12)	6.63 (2.86)	7.66 (1.91)	<0.001***
Follow-up	5.75 (3.40)	6.77 (3.07)	7.70 (2.28)	<0.001***
**CONF 6**				
Baseline	6.35 (3.26)	6.92 (2.84)	7.78 (2.04)	<0.001***
Follow-up	6.11 (3.37)	6.89 (3.03)	7.87 (2.23)	0.001**
**Participants who Returned for Follow-up**	**HCs (N=69)**	**DEP/ANX (N=135)**	**SUDs (N=83)**	***p****
**All trials**				
Baseline	6.90 (1.85)	7.26 (1.77)	7.58 (1.27)	0.044*
Follow-up	6.85 (2.00)	7.38 (1.90)	7.73 (1.54)	0.013*
**Approach**				
Baseline	8.65 (0.91)	8.24 (1.51)	7.98 (1.51)	0.013*
Follow-up	8.57 (1.12)	8.45 (1.28)	8.31 (1.29)	0.421
**Avoid**				
Baseline	8.29 (1.49)	7.91 (1.69)	6.44 (2.12)	<0.001***
Follow-up	8.29 (1.44)	8.21 (1.53)	7.25 (2.28)	<0.001***
**CONF 2**				
Baseline	5.46 (3.15)	6.33 (2.93)	7.53 (1.93)	<0.001***
Follow-up	5.53 (3.41)	6.60 (3.06)	7.53 (2.38)	<0.001***
**CONF 4**				
Baseline	5.91 (3.12)	6.79 (2.85)	7.89 (1.57)	<0.001***
Follow-up	5.75 (3.40)	6.77 (3.07)	7.70 (2.28)	<0.001***
**CONF 6**				
Baseline	6.19 (3.27)	7.02 (2.84)	8.05 ()	<0.001***
Follow-up	6.11 (3.37)	6.89 (3.03)	7.87 (2.23)	0.001**

Note: ****p* < 0.001, ***p* < 0.01, **p* < 0.05.

Supplementary Table 12. Summary statistics for variability (SD) in chosen runway positions (Mean (SD)) at baseline and follow-up

**Full Sample**	**HCs (N = 97)**	**DEP/ANX (N = 208)**	**SUDs (N = 175)**	***p****
**All trials**				
Baseline	2.09 (1.38)	1.99 (1.30)	2.03 (1.08)	0.795
Follow-up	2.22 (1.52)	1.70 (1.45)	1.71 (1.26)	0.033
**Approach**				
Baseline	0.61 (1.07)	0.78 (1.17)	1.13 (1.21)	0.001***
Follow-up	0.54 (1.12)	0.60 (1.04)	0.82 (1.17)	0.241
**Avoid**				
Baseline	0.83 (1.14)	0.95 (1.16)	1.76 (1.31)	<0.001***
Follow-up	0.71 (1.08)	0.70 (1.06)	1.28 (1.31)	0.001**
**CONF 2**				
Baseline	1.03 (1.12)	1.24 (1.23)	1.39 (1.35)	0.081
Follow-up	0.92 (1.22)	0.82 (1.12)	0.89 (1.13)	0.828
**CONF 4**				
Baseline	0.98 (1.15)	1.07 (1.23)	1.30 (1.30)	0.082
Follow-up	0.85 (1.24)	0.71 (1.01)	0.88 (1.19)	0.509
**CONF 6**				
Baseline	0.80 (1.15)	0.98 (1.19)	1.14 (1.35)	0.101
Follow-up	0.81 (1.27)	0.74 (1.10)	0.78 (1.23)	0.937
**Participants who Returned for Follow-up**	**HCs (N=69)**	**DEP/ANX (N=135)**	**SUDs (N=83)**	***p****
**All trials**				
Baseline	2.20(1.43)	1.97 (1.29)	2.08 ()	0.465
Follow-up	2.22(1.52)	1.70 (1.45)	1.71 (1.26)	0.033
**Approach**				
Baseline	0.46 (0.93)	0.80 (1.13)	1.19 (1.24)	<0.001***
Follow-up	0.54 (1.12)	0.60 (1.04)	0.82 (1.17)	0.241
**Avoid**				
Baseline	0.75 (1.13)	1.02 (1.19)	1.93 (1.26)	<0.001***
Follow-up	0.71 (1.08)	0.70 (1.06)	1.28 (1.31)	0.001**
**CONF 2**				
Baseline	1.04 (1.15)	1.20 (1.20)	1.39 (1.35)	0.220
Follow-up	0.92 (1.22)	0.82 (1.12)	0.89 (1.13)	0.828
**CONF 4**				
Baseline	1.06 (1.18)	0.95 (1.15)	1.28 (1.31)	0.138
Follow-up	0.85 (1.24)	0.71 (1.01)	0.88 (1.19)	0.509
**CONF 6**				
Baseline	0.85 (1.18)	0.88 (1.11)	1.14 (1.35)	0.231
Follow-up	0.81 (1.27)	0.74 (1.10)	0.78 (1.23)	0.937

Note: ***$p < 0.001$, **$p < 0.01$, *$p < 0.05$.

Supplementary Table 13. Results of linear mixed effects models predicting response time (RTs) in participants who returned for follow-up, when accounting for effects of group, time, and their interaction

Predictor:	Group	Time	Group x Time
**All Trials**	*F*(2, 283)=0.64, *p*=0.526	*F*(1, 283)=9.33, *p*=0.002<br><br>T1=1.257; T2=1.203<br><br>T1 - T2: *t*(283.21)=3.055, *p*=0.002, *d*=0.27	*F*(2, 283)=0.02, *p*=0.981
**Approach**	*F*(2, 283)=1.16, *p*=0.315	*F*(1, 283)=11.22, *p*<0.001<br><br>T1=1.235; T2=1.164<br><br>T1 - T2: *t*(283.23)=3.35, *p*<0.001, *d*=0.29	*F*(2, 283)=0.68, *p*=0.507
**Avoid**	*F*(2, 283)=9.54, *p*<0.001<br><br>D/A=1.336; HCs=1.282; SUDs=1.48<br><br>D/A - HCs: *t*(283.37)=1.218, *p*=0.224, *d*=0.21<br>D/A - SUDs: *t*(284.23)=-3.478, *p*<0.001, *d*=-0.58<br>HCs - SUDs: *t*(283.7)=-4.086, *p*<0.001, *d*=-0.79	*F*(1, 282)=2.89, *p*=0.090	*F*(2, 282)=1.32, *p*=0.269
**CONF2**	*F*(2, 283)=0.01, *p*=0.992	*F*(1, 283)=8.15, *p*=0.005<br><br>T1=1.243; T2=1.183<br><br>T1 - T2: *t*(283.24)=2.855, *p*=0.005, *d*=0.25	*F*(2, 283)=0.39, *p*=0.678
**CONF4**	*F*(2, 283)=0.09, *p*=0.912	*F*(1, 283)=6.64, *p*=0.010<br><br>T1=1.207; T2=1.152<br><br>T1 - T2: *t*(283.23)=2.577, *p*=0.010, *d*=0.22	*F*(2, 283)=0.33, *p*=0.723
**CONF6**	*F*(2, 283)=0.20, *p*=0.822	*F*(1, 283)=3.73, *p*=0.055	*F*(2, 283)=0.22, *p*=0.804

Note: HCs = Healthy Comparisons; D/A = Depression and Anxiety; SUDs = Substance Use Disorder; T1 = Baseline; T2 = Follow-up; NS = nonsignificant. For interpretability, sum coding was used for time (baseline = −1; follow-up = 1), and group (with HCs coded as -1).
****p* < 0.001, ***p* < 0.01, **p* < 0.05.

Supplementary Table 14. Results of linear mixed effects models predicting response time (RTs*)* in participants who returned for follow-up, when accounting for effects of group, time, age, and sex

Predictor:	Group	Time	Age	Sex	Group x Age	Group x Sex

**All Trials**	*F*(2, 278)=1.29, *p*=0.276	*F*(1, 290)=15.47, *p*<0.001<br><br>T1=1.26; T2=1.194<br><br>T1 - T2: *t*(290.61)=3.933, *p*<0.001, *d*=0.33	*F*(1, 278)=50.36, *p*<0.001<br><br>T1=1.26; T2=1.194	*F*(1, 278)=0.00, *p*=0.980	*F*(2, 283)=0.13, *p*=0.880	*F*(2, 278)=1.41, *p*=0.245
**Approach**	*F*(2, 278)=2.10, *p*=0.125	*F*(1, 289)=16.04, *p*<0.001<br><br>T1=1.238; T2=1.156<br><br>T1 - T2: *t*(289.99)=4.004, *p*<0.001, *d*=0.34	*F*(1, 278)=46.51, *p*<0.001<br><br>T1=1.238; T2=1.156	*F*(1, 278)=0.00, *p*=0.989	*F*(2, 282)=0.30, *p*=0.739	*F*(2, 278)=1.33, *p*=0.267
**Avoid**	*F*(2, 278)=9.81, *p*<0.001<br><br>D/A=1.33; HCs=1.269; SUDs=1.481<br><br>D/A - HCs: *t*(277.38)=1.543, *p*=0.124, *d*=0.25<br>D/A - SUDs: *t*(278.36)=-4.055,*p*<0.001, *d*=-0.6<br>HCs - SUDs: *t*(277.64)=-4.864, *p*<0.001, *d*=-0.85	*F*(1, 288)=7.53, *p* =0.006<br><br>T1=1.399; T2=1.341<br><br>T1 - T2: *t*(288.96)=2.744, *p*=0.006, *d*=0.23	*F*(1, 277)=58.58, *p*<0.001<br><br>T1=1.399; T2=1.341	*F*(1, 278)=1.17, *p*=0.281	*F*(2, 281)=0.08, *p*=0.919	*F*(2, 278)=4.20, *p*=0.016<br><br>Female EMM: D/A=1.351; HCs=1.224; SUDs=1.475;<br>Male EMM: D/A=1.272; HCs=1.401; SUDs=1.498<br><br>Female:<br>D/A - HCs: *t*(276.98)=2.71, *p*=0.007, *d*=0.51<br>D/A - SUDs: *t*(278.41)=-2.89, *p*=0.004, *d*=-0.5<br>HCs - SUDs: *t*(277.94)=-4.824, *p*<0.001, *d*=-1.01<br><br>Male:<br>D/A - HCs: *t*(278.62)=-1.726, *p*=0.085, *d*=-0.52<br>D/A - SUDs: *t*(278.73)=-2.949, *p*=0.003, *d*=-0.91<br>HCs - SUDs: *t*(277.08)=-1.215,

						p=0.225, *d*=-0.39
**CONF2**	*F*(2, 278)=0.41, *p*=0.667	*F*(1, 289)=11.65, *p*<0.001<br><br>T1=1.242; T2=1.172<br><br>T1 - T2: *t*(290)=3.414, *p*<0.001, *d*=0.29	*F*(1, 278)=36.45, *p*<0.001<br><br>T1=1.242; T2=1.172	*F*(1, 278)=0.30, *p*=0.585	*F*(2, 282)=0.02, *p*=0.983	*F*(2, 278)=0.87, *p*=0.418
**CONF4**	*F*(2, 278)=0.05, *p*=0.948	*F*(1, 290)=10.73, *p*=0.001<br><br>T1=1.209; T2=1.142<br><br>T1 - T2: *t*(290.34)=3.275, *p*=0.001, *d*=0.27	*F*(1, 278)=30.28, *p*<0.001<br><br>T1=1.209; T2=1.142	*F*(1, 278)=0.07, *p*=0.799	*F*(2, 282)=0.18, *p*=0.831	*F*(2, 278)=0.80, *p*=0.449
**CONF6**	*F*(2, 278)=0.38, *p*=0.684	*F*(1, 289)=6.18, *p*=0.014<br><br>T1=1.214; T2=1.162<br><br>T1 - T2: *t*(289.56)=2.485, *p*=0.014, *d*=0.21	*F*(1, 278)=29.88, *p*<0.001<br><br>T1=1.214; T2=1.162	*F*(1, 278)=0.11, *p*=0.746	*F*(2, 281)=0.06, *p*=0.945	*F*(2, 278)=0.36, *p*=0.696

Note: HCs = Healthy Comparisons; D/A = Depression and Anxiety; SUDs = Substance Use Disorder; T1 = Baseline; T2 = Follow-up; NS = nonsignificant. For interpretability, age was centered, and sum coding was used for sex (female = −1; male = 1), time (baseline = −1; follow-up = 1), and group (with HCs coded as -1).

Supplementary Table 15. Results of linear mixed effects models predicting response times (RTs) in participants who returned for follow-up, when accounting for effects of group, time, age, and sex

**Predictor:**	**Group**	**Time**	**Age**	**Sex**	**WRAT**	**Group x Age**	**Group x Sex**	**Group x WRAT**
**All Trials**	*F*(2, 232)=0.70, *p*=0.499	*F*(1, 243)=12.28, *p*<0.001<br><br>T1=1.256; T2=1.192<br>T1 - T2: *t*(243.9)=3.504, *p*<0.001,	*F*(1, 231)=44.26, *p*<0.001<br><br>T1=1.256; T2=1.192	*F*(1, 231)=0.40, *p*=0.530	*F*(1, 232)=4.07, *p*=0.045	*F*(2, 235)=0.54, *p*=0.583	*F*(2, 231)=2.39, *p*=0.094	*F*(2, 233)=0.69, *p*=0.504

		$d=0.32$						
**Approach**	$F(2, 232)=0.67$, $p=0.515$	$F(1, 243)=11.40$, $p<0.001$<br><br>T1=1.228; T2=1.154<br><br>T1 - T2: $t(243.61)=3.376$, $p<0.001$, $d=0.31$	$F(1, 231)=42.16$, $p<0.001$<br><br>T1=1.228; T2=1.154	$F(1, 230)=0.07$, $p=0.788$	$F(1, 232)=3.13$, $p=0.078$	$F(2, 234)=0.62$, $p=0.538$	$F(2, 231)=1.55$, $p=0.215$	$F(2, 233)=0.55$, $p=0.580$
**Avoid**	$F(2, 231)=4.58$, $p=0.011$<br><br>D/A=1.337; HCs=1.261; SUDs=1.443<br><br>D/A - HCs: $t(231.72)=1.679$, $p=0.095$, $d=0.31$<br>D/A - SUDs: $t(233)=-2.329$, $p=0.021$, $d=-0.44$<br>HCs - SUDs: $t(230.11)=-3.464$, $p<0.001$, $d=-0.76$	$F(1, 243)=7.47$, $p=0.007$<br><br>T1=1.401; T2=1.341<br><br>T1 - T2: $t(243.55)=2.733$, $p=0.007$, $d=0.25$	$F(1, 231)=44.89$, $p<0.001$<br><br>T1=1.401; T2=1.341	$F(1, 230)=3.52$, $p=0.062$	$F(1, 232)=8.58$, $p=0.004$	$F(2, 234)=0.13$, $p=0.880$	$F(2, 231)=4.70$, $p=0.010$<br><br>Female EMM:<br>D/A=1.353; HCs=1.216; SUDs=1.417<br>Male EMM:<br>D/A=1.276; HCs=1.426; SUDs=1.539<br><br>Female:<br>D/A - HCs: $t(231.03)=2.663$, $p=0.008$, $d=0.57$<br>D/A - SUDs: $t(232.8)=-1.227$, $p=0.221$ $d=-0.26$<br>HCs - SUDs: $t(230.37)=-3.323$, $p=0.001$, $d=-0.84$<br>Male:<br>D/A - HCs: $t(233.2)=-1.792$, $p=0.074$, $d=-0.62$<br>D/A - SUDs: $t(231.96)=-2.593$, $p=0.010$, $d=-1.09$	$F(2, 233)=0.05$, $p=0.949$

							HCs - SUDs: $t(229.12)=-1.08$, $p=0.281$, $d=-0.47$	
**CON F2**	$F(2, 232)=0.75$, $p=0.472$	$F(1, 243)=9.50$, $p=0.002$<br><br>T1=1.232; T2=1.165<br><br>T1 - T2: $t(243.76)=3.082$, $p=0.002$, $d=0.28$	$F(1, 231)=33.69$, $p<0.001$<br><br>T1=1.232; T2=1.165	$F(1, 231)=0.00$, $p=0.973$	$F(1, 232)=1.04$, $p=0.309$	$F(2, 235)=0.18$, $p=0.832$	$F(2, 231)=1.60$, $p=0.203$	$F(2, 233)=1.07$, $p=0.345$
**CON F4**	$F(2, 232)=0.38$, $p=0.684$	$F(1, 243)=6.84$, $p=0.009$<br><br>T1=1.207; T2=1.15<br><br>T1 - T2: $t(243.75)=2.615$, $p=0.009$, $d=0.24$	$F(1, 231)=29.52$, $p<0.001$<br><br>T1=1.207; T2=1.15	$F(1, 231)=0.62$, $p=0.433$	$F(1, 232)=1.80$, $p=0.181$	$F(2, 235)=1.07$, $p=0.345$	$F(2, 231)=2.70$, $p=0.069$	$F(2, 233)=1.07$, $p=0.345$
**CON F6**	$F(2, 231)=1.06$, $p=0.348$	$F(1, 243)=6.18$, $p=0.014$<br><br>T1=1.208; T2=1.152<br><br>T1 - T2: $t(243.47)=2.486$, $p=0.014$, $d=0.23$	$F(1, 231)=23.56$, $p<0.001$<br><br>T1=1.208; T2=1.152	$F(1, 230)=0.01$, $p=0.927$	$F(1, 232)=4.03$, $p=0.046$	$F(2, 234)=0.41$, $p=0.663$	$F(2, 231)=0.57$, $p=0.568$	$F(2, 232)=0.77$, $p=0.462$

Note: HCs = Healthy Comparisons; D/A = Depression and Anxiety; SUDs = Substance Use Disorder; T1 = Baseline; T2 = Follow-up; NS = nonsignificant.
For interpretability, age and WRAT scores were centered, and sum coding was used for sex (female = −1; male = 1), time (baseline = −1; follow-up = 1), and group (with HCs coded as -1).

Supplementary Table 16. Results of linear mixed effects models predicting average chosen runway position in participants who returned for follow-up, when accounting for effects of group, time, and their interaction

Predictor:	Group	Time	Group x Time
**All Trials**	*F*(2, 284)=4.47, *p*=0.012<br><br>D/A=7.321; HCs=6.874; SUDs=7.656<br><br>D/A - HCs: *t*(284)=1.88, *p*=0.061, *d*=0.46<br>D/A - SUDs: *t*(284)=-1.498, *p*=0.135, *d*=-0.34<br>HCs - SUDs: *t*(284)=-2.99, *p*=0.003, *d*=-0.8	*F*(1, 284)=0.83, *p*=0.362	*F*(2, 283)=0.48, *p*=0.617
**Approach**	*F*(2, 283)=3.08, *p*=0.048<br><br>D/A=8.348; HCs=8.613; SUDs=8.146<br><br>D/A - HCs: *t*(284)=-1.546, *p*=0.123, *d*=-0.3<br>D/A - SUDs: *t*(284)=1.255, *p*=0.210, *d*=0.23<br>HCs - SUDs: *t*(284)=2.479, *p*=0.014, *d*=0.52	*F*(1, 284)=3.99, *p*=0.047<br><br>T1=8.291; T2=8.447<br><br>T1 - T2: *t*(284)=-1.999, *p*=0.047, *d*=-0.17	*F*(2, 284)=2.03, *p*=0.133
**Avoid**	*F*(2, 284)=20.14, *p*<0.001<br><br>D/A=8.056; HCs=8.29; SUDs=6.847<br><br>D/A - HCs: *t*(284)=-1.002, *p*=0.317, *d*=-0.21<br>D/A - SUDs: *t*(284)=5.49, *p*<0.001, *d*=1.06<br>HCs - SUDs: *t*(284)=5.61, *p*<0.001, *d*=1.27	*F*(1, 284)=14.22, *p*<0.001<br><br>T1=7.545; T2=7.917<br><br>T1 - T2: *t*(284)=-3.771, *p*<0.001, *d*=-0.33	*F*(2, 283)=5.04, *p*=0.007<br><br><u>Baseline EMM</u>:<br>D/A=7.906; HCs=8.287; SUDs=6.441;<br><u>Follow-up EMM</u>:<br>D/A=8.206; HCs=8.292; SUDs=7.253<br><br><u>Baseline:</u><br>D/A - HCs: *t*(422.13)=-1.456, *p*=0.146, *d*=-0.34<br>D/A - SUDs: *t*(422.13)=5.927, *p*<0.001, *d*=1.29<br>HCs - SUDs: *t*(422.13)=6.397, *p*<0.001, *d*=1.62<br><u>Follow-up:</u><br>D/A - HCs: *t*(422.13)=-0.328, *p*=0.743, *d*=-0.08<br>D/A - SUDs: *t*(422.13)=3.857, *p*<0.001, *d*=0.84<br>HCs - SUDs: *t*(422.13)=3.6, *p*<0.001, *d*=0.91
**CONF2**	*F*(2, 283)=11.57, *p*<0.001<br><br>D/A=6.462; HCs=5.492; SUDs=7.533<br><br>D/A - HCs: *t*(284)=2.506, *p*=0.013, *d*=0.59<br>D/A - SUDs: *t*(284)=-2.933, *p*=0.004, *d*=-0.65	*F*(1, 283)=0.64, *p*=0.426	*F*(2, 284)=0.41, *p*=0.667

	HCs - SUDs: $t(284)=-4.788$, $p<0.001$ $d=-1.25$		
**CONF4**	$F(2, 284)=11.29$, $p<0.001$<br><br>D/A=6.783; HCs=5.825; SUDs=7.796<br><br>D/A - HCs: $t(284)=2.533$, $p=0.012$, $d=0.6$<br>D/A - SUDs: $t(284)=-2.844$, $p=0.005$, $d=-0.64$<br>HCs - SUDs: $t(284)=-4.736$, $p<0.001$ $d=-1.24$	$F(1, 283)=0.78$, $p=0.379$	$F(2, 283)=0.18$, $p=0.836$
**CONF6**	$F(2, 284)=9.54$, $p<0.001$<br><br>D/A=6.954; HCs=6.148; SUDs=7.96<br><br>D/A - HCs: $t(284)=2.118$, $p=0.035$, $d=0.52$<br>D/A - SUDs: $t(284)=-2.805$, $p=0.005$, $d=-0.65$<br>HCs - SUDs: $t(284)=-4.325$, $p<0.001$, $d=-1.18$	$F(1, 283)=1.00$, $p=0.318$	$F(2, 284)=0.05$, $p=0.954$

Note: HCs = Healthy Comparisons; D/A = Depression and Anxiety; SUDs = Substance Use Disorder; T1 = Baseline; T2 = Follow-up; NS = nonsignificant. For interpretability, sum coding was used for time (baseline = −1; follow-up = 1), and group (with HCs coded as -1).

Supplementary Table 17. Results of linear mixed effects models predicting average chosen runway positions in participants who returned for follow-up, when accounting for effects of group, time, age, and sex

**Predictor:**	**Group**	**Time**	**Age**	**Sex**	**Group x Age**	**Group x Sex**
**All Trials**	$F(2, 278)=2.99$, $p=0.052$	$F(1, 295)=2.10$, $p=0.149$	$F(1, 279)=4.93$, $p=0.027$	$F(1, 278)=0.93$, $p=0.336$	$F(2, 287)=0.68$, $p=0.507$	$F(2, 278)=1.67$, $p=0.190$
**Approach**	$F(2, 278)=3.87$, $p=0.022$<br><br>D/A=8.335; HCs=8.598; SUDs=8.142<br><br>D/A - HCs: $t(278)=-1.552$, $p=0.122$, $d=-0.29$<br>D/A - SUDs: $t(278)=1.224$, $p=0.222$, $d=0.22$	$F(1, 291)=7.25$, $p=0.007$<br><br>T1=8.234; T2=8.437<br>T1 - T2: $t(291.59)=-2.693$, $p=0.007$, $d=-0.23$	$F(1, 279)=10.07$, $p=0.002$<br><br>T1=8.234; T2=8.437	$F(1, 278)=0.36$, $p=0.550$	$F(2, 283)=0.54$, $p=0.585$	$F(2, 278)=1.14$, $p=0.321$

	HCs - SUDs: *t*(278)=2.457, *p*=0.015, *d*=0.51					
**Avoid**	*F*(2, 278)=12.76, *p*<0.001<br><br>D/A=8.054; HCs=8.3; SUDs=6.842<br><br>D/A - HCs: *t*(278)=-1.048, *p*=0.296, *d*=-0.21<br>D/A - SUDs: *t*(278)=5.514, *p*<0.001, *d*=1.05<br>HCs - SUDs: *t*(278)=5.654, *p*<0.001, *d*=1.26	*F*(1, 292)=17.14, *p*<0.001<br><br>T1=7.545; T2=7.946<br><br>T1 - T2: *t*(292.57)=-4.141, *p*<0.001, *d*=-0.35	*F*(1, 279)=3.86, *p*=0.051	*F*(1, 278)=0.06, *p*=0.799	*F*(2, 284)=0.42, *p*=0.658	*F*(2, 278)=0.79, *p*=0.457
**CONF2**	*F*(2, 278)=7.46, *p*<0.001<br><br>D/A=6.453; HCs=5.396; SUDs=7.526<br><br>D/A - HCs: *t*(278)=2.712, *p*=0.007, *d*=0.65<br>D/A - SUDs: *t*(278)=-2.941, *p*=0.004, *d*=-0.66<br>HCs - SUDs: *t*(278)=-4.978, *p*<0.001, *d*=-1.31	*F*(1, 295)=1.50, *p*=0.222	*F*(1, 279)=1.40, *p*=0.237	*F*(1, 278)=0.73, *p*=0.393	*F*(2, 287)=0.87, *p*=0.419	*F*(2, 278)=1.78, *p*=0.170
**CONF4**	*F*(2, 278)=7.98, *p*<0.001<br><br>D/A=6.774; HCs=5.738; SUDs=7.789<br><br>D/A - HCs: *t*(278)=2.718, *p*=0.007, *d*=0.65<br>D/A - SUDs: *t*(278)=-2.844, *p* = 0.005 *d*=-0.64<br>HCs - SUDs: *t*(278)=-4.901, *p*<0.001, *d*=-1.29	*F*(1, 295)=0.31, *p*=0.580	*F*(1, 279)=2.08, *p*=0.151	*F*(1, 278)=0.93, *p*=0.335	*F*(2, 287)=0.31, *p*=0.736	*F*(2, 278)=1.46, *p*=0.235

**CONF6**	$F(2, 278)=7.18$, $p<0.001$<br><br>D/A=6.952; HCs=6.063; SUDs=7.95<br><br>D/A - HCs: $t(278)=2.317$, $p=0.021$, $d=0.58$<br>D/A - SUDs: $t(278)=-2.782$, $p=0.006$, $d=-0.65$<br>HCs - SUDs: $t(278)=-4.483$, $p<0.001$, $d=-1.23$	$F(1, 295)=0.66$, $p=0.416$	$F(1, 279)=2.81$, $p=0.095$	$F(1, 278)=1.65$, $p=0.200$	$F(2, 288)=0.45$, $p=0.639$	$F(2, 278)=1.10$, $p=0.336$

Note: HCs = Healthy Comparisons; D/A = Depression and Anxiety; SUDs = Substance Use Disorder; T1 = Baseline; T2 = Follow-up; NS = nonsignificant.
For interpretability, Age was centered, and sum coding was used for sex (female = −1; male = 1), time (baseline = −1; follow-up = 1), and group (with HCs coded as -1).

Supplementary Table 18. Results of linear mixed effects models predicting average chosen runway positions in participants who returned for follow-up, when accounting for effects of group, time, age, sex, and WRAT scores

**Predictor:**	**Group**	**Time**	**Age**	**Sex**	**WRAT**	**Group x Age**	**Group x Sex**	**Group x WRAT**
**All Trials**	$F(2, 232)=4.52$, $p=0.012$<br><br>D/A=7.44; HCs=6.585; SUDs=7.826<br><br>D/A - HCs: $t(232.47)=3.26$, $p=0.001$, $d=0.94$<br>D/A - SUDs: $t(233.23)=-1.455$, $p=0.147$, $d=-0.43$<br>HCs - SUDs: $t(231.53)=-4.057$, $p<0.001$, $d=-1.37$	$F(1, 246)=2.81$, $p=0.095$	$F(1, 233)=8.90$, $p=0.003$	$F(1, 232)=3.01$, $p=0.084$	$F(1, 233)=0.17$, $p=0.680$	$F(2, 241)=1.62$, $p=0.200$	$F(2, 232)=1.56$, $p=0.212$	$F(2, 233)=1.28$, $p=0.279$
**Approach**	$F(2, 232)=1.89$, $p=0.153$	$F(1, 244)=6.94$, $p=0.009$<br><br>T1=8.286; T2=8.491<br><br>T1 - T2:	$F(1, 232)=9.95$, $p=0.002$<br><br>T1=8.286; T2=8.4	$F(1, 231)=0.00$, $p=0.970$	$F(1, 232)=14.30$, $p<0.001$	$F(2, 236)=1.04$, $p=0.355$	$F(2, 231)=1.69$, $p=0.187$	$F(2, 233)=1.12$, $p=0.329$

		$t(244.04)=-2.634$,<br>$p=0.009$,<br>$d=-0.24$	91					
**Avoid**	$F(2, 232)=5.68$,<br>$p=0.004$<br><br>D/A=7.863;<br>HCs=8.161;<br>SUDs=6.813<br><br>D/A - HCs:<br>$t(232.05)=-1.157$,<br>$p=0.249$, $d=-0.25$<br>D/A - SUDs:<br>$t(233.15)=4.035$,<br>$p<0.001$, $d=0.9$<br>HCs - SUDs:<br>$t(230.69)=4.495$,<br>$p<0.001$, $d=1.15$	$F(1, 244)=14.50$, $p<0.001$<br><br>T1 =7.443;<br>T2= 7.854<br><br>T1 - T2:<br>$t(244.2)=-3.807$,<br>$p<0.001$,<br>$d=-0.35$	$F(1, 232)=3.80$,<br>$p=0.053$	$F(1, 231)=0.21$,<br>$p=0.645$	$F(1, 232)=18.71$,<br>$p<0.001$	$F(2, 236)=0.85$,<br>$p=0.430$	$F(2, 231)=0.38$, $p=0.686$	$F(2, 233)=5.58$,<br>$p=0.004$<br><br>D/A = 0.131; HCs = 0.098; SUDs = 0.004<br><br>D/A - HCs:<br>$t(234)=0.712$, p = 0.757<br>D/A - SUDs:<br>$t(235)=3.24$, $p = 0.004$, $d=0.11$<br>HCs - SUDs:<br>$t(230)=2.06$,<br>$p=0.101$
**CONF2**	$F(2, 232)=7.41$,<br>$p<0.001$<br><br>D/A=6.787;<br>HCs=5.112;<br>SUDs=7.792<br><br>D/A - HCs:<br>$t(232.44)=3.905$,<br>$p<0.001$, $d=1.1$<br>D/A - SUDs:<br>$t(233.23)=-2.318$,<br>$p=0.021$, $d=-0.66$<br>HCs - SUDs:<br>$t(231.47)=-5.358$,<br>$p<0.001$, $d=-1.77$	$F(1, 246)=1.58$,<br>$p=0.210$	$F(1, 233)=3.70$,<br>$p=0.056$	$F(1, 231)=3.07$,<br>$p=0.081$	$F(1, 233)=1.93$,<br>$p=0.167$	$F(2, 240)=1.59$,<br>$p=0.205$	$F(2, 232)=1.80$, $p=0.167$	$F(2, 233)=2.72$,<br>$p=0.068$
**CONF4**	$F(2, 232)=7.62$,<br>$p<0.001$<br><br>D/A=7.054;<br>HCs=5.444;<br>SUDs=8.003<br><br>D/A - HCs:<br>$t(232.44)=3.825$,<br>$p<0.001$, $d=1.08$<br>D/A - SUDs:	$F(1, 246)=0.08$,<br>$p=0.773$	$F(1, 233)=5.35$,<br>$p=0.022$	$F(1, 231)=2.44$,<br>$p=0.120$	$F(1, 233)=0.66$,<br>$p=0.419$	$F(2, 240)=1.13$,<br>$p=0.324$	$F(2, 232)=1.31$, $p=0.272$	$F(2, 233)=2.12$,<br>$p=0.123$

	$t(233.23)=-2.231$, $p=0.027$, $d=-0.64$<br>HCs - SUDs: $t(231.48)=-5.214$, $p<0.001$, $d=-1.72$							
**CONF6**	$F(2, 232)=6.55$, $p=0.002$<br><br>D/A=7.202; HCs=5.747; SUDs=8.129<br><br>D/A - HCs: $t(232.5)=3.413$, $p<0.001$, $d=1.02$<br>D/A - SUDs: $t(233.23)=-2.152$, $p=0.032$, $d=-0.65$<br>HCs - SUDs: $t(231.61)=-4.791$, $p<0.001$, $d=-1.67$	$F(1, 247)=0.15$, $p=0.696$	$F(1, 233)=6.13$, $p=0.014$	$F(1, 232)=3.03$, $p=0.083$	$F(1, 233)=0.61$, $p=0.435$	$F(2, 242)=0.93$, $p=0.394$	$F(2, 232)=1.14$, $p=0.322$	$F(2, 233)=1.99$, $p=0.139$

Note: HCs = Healthy Comparisons; D/A = Depression and Anxiety; SUDs = Substance Use Disorder; T1 = Baseline; T2 = Follow-up; NS = nonsignificant. For interpretability, age and WRAT scores were centered, and sum coding was used for sex (female = −1; male = 1), time (baseline = −1; follow-up = 1), and group (with HCs coded as -1).

Supplementary Table 19. Results of linear mixed effects models predicting choice variability in participants who returned for follow-up, when accounting for effects of group, time, and their interaction

**Predictor:**	**Group**	**Time**	**Group x Time**
**All Trials**	$F(2, 283)=2.28$, $p=0.104$	$F(1, 284)=8.28$, $p=0.004$<br><br>T1=2.081; T2=1.874<br><br>T1 - T2: $t(284)=2.877$, $p=0.004$, $d=0.25$	$F(2, 284)=2.25$, $p=0.107$
**Approach**	$F(2, 284)=5.89$, $p=0.003$<br><br>D/A=0.701; HCs=0.501; SUDs=1.006<br><br>D/A - HCs: $t(284)=1.464$, $p=0.144$, $d=0.23$<br>D/A - SUDs: $t(284)=-2.371$, $p=0.018$, $d=-0.35$<br>HCs - SUDs: $t(284)=-3.361$, $p<0.001$, $d=-0.57$	$F(1, 284)=4.62$, $p=0.033$<br><br>T1=0.818; T2=0.654<br><br>T1 - T2: $t(284)=2.149$, $p=0.033$, $d=0.19$	$F(2, 284)=2.49$, $p=0.084$

**Avoid**	*F*(2, 284)=18.52, *p*<0.001,<br><br>D/A=0.859; HCs=0.731; SUDs=1.605<br><br>D/A - HCs: *t*(284)=0.864, *p*=0.388, *d*=0.15<br>D/A - SUDs: *t*(284)=-5.313, *p*<0.001, *d*=-0.88<br>HCs - SUDs: *t*(284)=-5.334, *p*<0.001, *d*=-1.03	*F*(1, 284)=21.24, *p*<0.001<br><br>T1=1.235; T2=0.895<br><br>T1 - T2: *t*(284)=4.609, *p*<0.001, *d*=0.4	*F*(2, 284)=5.03, *p*=0.007<br><br>Baseline EMM:<br>D/A=1.018; HCs=0.753; SUDs=1.935;<br>Follow-up EMM:<br>D/A=0.701; HCs=0.708; SUDs=1.276<br><br>Baseline:<br>D/A - HCs: *t*(463.59)=1.524, *p*=0.128, *d*=0.31<br>D/A - SUDs: *t*(463.59)=-5.611, *p*<0.001, *d*=-1.08<br>HCs - SUDs: *t*(463.59)=-6.188, *p*<0.001, *d*=-1.39<br><br>Follow-up:<br>D/A - HCs: *t*(463.59)=-0.04, *p*=0.968, *d*=-0.01<br>D/A - SUDs: *t*(463.59)=-3.513, *p*<0.001, *d*=-0.68<br>HCs - SUDs: *t*(463.59)=-2.971, *p*=0.003, *d*=-0.67
**CONF2**	*F*(2, 284)=0.63, *p*=0.533	*F*(1, 283)=16.49, *p*<0.001<br><br>T1=1.211; T2=0.877<br><br>T1 - T2: *t*(284)=4.061, *p*<0.001, *d*=0.35	*F*(2, 283)=1.49, *p*=0.226
**CONF4**	*F*(2, 284)=1.76, *p*=0.173	*F*(1, 283)=12.76, *p*<0.001<br><br>T1=1.097; T2=0.811<br><br>T1 - T2: *t*(284)=3.572, *p*<0.001, *d*=0.31	*F*(2, 284)=0.56, *p*=0.570
**CONF6**	*F*(2, 284)=0.60, *p*=0.551	*F*(1, 284)=5.04, *p*=0.026<br><br>T1=0.959; T2=0.776<br><br>T1 - T2: *t*(284)=2.245, *p*=0.026, *d*=0.19	*F*(2, 283)=1.19, *p*=0.307

Note: HCs = Healthy Comparisons; D/A = Depression and Anxiety; SUDs = Substance Use Disorder; T1 = Baseline; T2 = Follow-up; NS = nonsignificant.

For interpretability, sum coding was used for time (baseline = −1; follow-up = 1), and group (with HCs coded as -1).

Supplementary Table 20. Results of linear mixed effects models predicting choice variability in participants who returned for follow-up, when accounting for effects of group, time, age, and sex

Predictor:	Group	Time	Age	Sex	Group x Age	Group x Sex
**All Trials**	*F*(2, 278)=1.38, *p*=0.254	*F*(1, 293)=12.53, *p*<0.001<br><br>T1=2.066; T2=1.819<br><br>T1 - T2: *t*(293.33)=3.539, *p*<0.001, *d*=0.3	*F*(1, 279)=3.72, *p*=0.055	*F*(1, 278)=1.17, *p*=0.280	*F*(2, 285)=0.78, *p*=0.458	*F*(2, 278)=0.34, *p*=0.715
**Approach**	*F*(2, 278)=6.56, *p*=0.002<br><br>D/A=0.698; HCs=0.515; SUDs=1.009<br><br>D/A - HCs: *t*(278)=1.366, *p*=0.173, *d*=0.21<br>D/A - SUDs: *t*(278)=-2.472, *p*=0.014, *d*=-0.35<br>HCs - SUDs: *t*(278)=-3.352, *p*<0.001, *d*=-0.56	*F*(1, 289)=7.72, *p*=0.006<br><br>T1=0.843; T2=0.637<br><br>T1 - T2: *t*(289.66)=2.779, *p*=0.006, *d*=0.23	*F*(1, 278)=14.77, *p*<0.001	*F*(1, 278)=0.00, *p*=0.977	*F*(2, 281)=0.49, *p*=0.615	*F*(2, 278)=0.45, *p*=0.636
**Avoid**	*F*(2, 278)=16.13, *p*<0.001<br><br>D/A=0.86; HCs=0.712; SUDs=1.605<br><br>D/A - HCs: *t*(278)=1.005, *p*=0.316, *d*=0.17<br>D/A - SUDs: *t*(278)=-5.396, *p*<0.001, *d*=-0.86<br>HCs - SUDs:	*F*(1, 290)=26.39, *p*<0.001<br><br>T1=1.296; T2=0.925<br><br>T1 - T2: *t*(290.65)=5.137, *p*<0.001, *d*=0.43	*F*(1, 278)=7.80, *p*=0.006<br><br>T1=1.296; T2=0.925	*F*(1, 278)=2.31, *p*=0.130	*F*(2, 282)=0.75, *p*=0.471	*F*(2, 278)=0.17, *p*=0.841

	$t(278)=-5.514$,<br>$p<0.001$, $d=-1.04$					
**CONF 2**	$F(2, 278)=0.77$,<br>$p=0.465$	$F(1, 289)=21.86$,<br>$p<0.001$<br><br>T1=1.221;<br>T2=0.85<br><br>T1 - T2:<br>$t(289.78)=4.676$, $p<0.001$,<br>$d=0.39$	$F(1, 278)=7.90$,<br>$p=0.005$	$F(1, 278)=0.08$, $p=0.772$	$F(2, 281)=1.33$,<br>$p=0.267$	$F(2, 278)=0.02$,<br>$p=0.975$
**CONF 4**	$F(2, 278)=2.18$,<br>$p=0.115$	$F(1, 289)=14.52$,<br>$p<0.001$<br><br>T1=1.092;<br>T2=0.798<br><br>T1 - T2:<br>$t(289.86)=3.811$, $p<0.001$,<br>$d=0.32$	$F(1, 278)=3.58$,<br>$p=0.059$	$F(1, 278)=0.08$, $p=0.781$	$F(2, 282)=1.30$,<br>$p=0.275$	$F(2, 278)=0.25$,<br>$p=0.779$
**CONF 6**	$F(2, 278)=0.56$,<br>$p=0.574$	$F(1, 289)=6.23$,<br>$p=0.013$<br><br>T1=0.948;<br>T2=0.751<br><br>T1 - T2:<br>$t(289.86)=2.496$, $p=0.013$,<br>$d=0.21$	$F(1, 278)=4.72$,<br>$p=0.031$<br><br>T1=0.948;<br>T2=0.751	$F(1, 278)=0.46$, $p=0.498$	$F(2, 282)=1.78$,<br>$p=0.171$	$F(2, 278)=0.08$,<br>$p=0.927$

Note: HCs = Healthy Comparisons; D/A = Depression and Anxiety; SUDs = Substance Use Disorders; T1 = Baseline; T2 = Follow-up; NS = nonsignificant. For interpretability, age was centered, and sum coding was used for sex (female = −1; male = 1), time (baseline = −1; follow-up = 1), and group (with HCs coded as -1).

Supplementary Table 21. Results of linear mixed effects models predicting choice variability in participants who returned for follow-up, when accounting for effects of group, time, age, sex and WRAT scores

Predictor:	Group	Time	Age	Sex	WRAT	Group x Age	Group x Sex	Group x WRAT

**All Trials**	$F(2, 232)=3.19$, $p=0.043$<br><br>D/A=1.773; HCs=2.384; SUDs=1.799<br><br>D/A - HCs: $t(232.24)=-3.064$, $p=0.002$, $d=-0.75$<br>D/A - SUDs: $t(233.2)=-0.127$, $p=0.899$, $d=-0.03$<br>HCs - SUDs: $t(231.07)=2.518$, $p=0.012$, $d=0.72$	$F(1, 245)=12.73$, $p< 0.001$<br>T1=2.006; T2=1.739<br><br>T1 - T2: $t(245.03)=3.567$, $p<0.001$, $d=0.33$	$F(1, 232)=6.59$, $p=0.011$	$F(1, 231)=3.78$, $p=0.053$	$F(1, 233)=0.58$, $p=0.445$	$F(2, 238)=2.01$, $p=0.137$	$F(2, 232)=0.11$, $p=0.900$	$F(2, 233)=0.63$, $p=0.536$
**Approach**	$F(2, 231)=0.82$, $p=0.443$	$F(1, 243)=4.37$, $p=0.038$<br><br>T1=0.767; T2=0.602<br><br>T1 - T2: $t(243.37)=2.091$, $p=0.038$, $d=0.19$	$F(1, 231)=13.11$, $p<0.001$<br><br>T1=0.767; T2=0.602	$F(1, 230)=0.80$, $p=0.373$	$F(1, 232)=16.28$, $p<0.001$	$F(2, 233)=0.56$, $p=0.574$	$F(2, 231)=0.22$, $p=0.802$	$F(2, 232)=1.65$, $p=0.194$
**Avoid**	$F(2, 231)=5.24$, $p=0.006$<br><br>D/A=0.962; HCs=0.753; SUDs=1.475<br><br>D/A - HCs: $t(231.64)=1.306$, $p=0.193$, $d=0.24$<br>D/A - SUDs: $t(232.96)=-3.155$, $p=0.002$, $d=-0.58$<br>HCs - SUDs: $t(230)=-3.862$, $p<0.001$, $d=-0.82$	$F(1, 243)=21.42$, $p<0.001$<br><br>T1=1.287; T2=0.912<br><br>T1 - T2: $t(243.48)=4.629$, $p<0.001$, $d=0.42$	$F(1, 231)=5.95$, $p=.015$<br><br>T1=1.287; T2=0.912	$F(1, 230)=0.69$, $p=0.406$	$F(1, 232)=16.10$, $p<0.001$	$F(2, 234)=1.47$, $p=0.232$	$F(2, 231)=0.43$, $p=0.651$	$F(2, 232)=1.51$, $p=0.223$
**CONF2**	$F(2, 231)=0.20$, $p=0.817$	$F(1, 243)=13.15$, $p<0.001$<br><br>T1=1.201; T2=0.88	$F(1, 230)=6.03$, $p=0.015$	$F(1, 230)=0.27$, $p=0.607$	$F(1, 232)=10.09$, $p=0.002$	$F(2, 233)=0.52$, $p=0.595$	$F(2, 230)=0.03$, $p=0.971$	$F(2, 232)=1.78$, $p=0.171$

		T1 - T2: $t(243.34)=3.626$, $p<0.001$, $d=0.33$						
**CON F4**	$F(2, 231)=0.85$, $p=0.428$	$F(1, 243)=13.98$, $p<0.001$<br><br>T1=1.126; T2=0.811<br><br>T1 - T2: $t(243.41)=3.738$, $p<0.001$, $d=0.34$	$F(1, 231)=2.68$, $p=0.103$	$F(1, 230)=0.04$, $p=0.833$	$F(1, 232)=8.05$, $p=0.005$	$F(2, 234)=1.11$, $p=0.331$	$F(2, 231)=0.41$, $p=0.662$	$F(2, 232)=1.63$, $p=0.199$
**CON F6**	$F(2, 231)=0.08$, $p=0.921$	$F(1, 243)=4.58$, $p=0.033$<br><br>T1=0.967; T2=0.781<br><br>T1 - T2: $t(243.36)=2.139$, $p=0.033$, $d=0.2$	$F(1, 230)=3.54$, $p=0.061$	$F(1, 230)=0.46$, $p=0.497$	$F(1, 232)=10.89$, $p=0.001$	$F(2, 233)=0.96$, $p=0.385$	$F(2, 231)=0.04$, $p=0.963$	$F(2, 232)=1.37$, $p=0.255$

Note: HCs = Healthy Comparisons; D/A = Depression and Anxiety; SUDs = Substance Use Disorder; T1 = Baseline; T2 = Follow-up; NS = nonsignificant.
For interpretability, age and WRAT scores were centered, and sum coding was used for sex (female = −1; male = 1), time (baseline = −1; follow-up = 1), and group (with HCs coded as -1).

Supplementary Table 22. Results of linear mixed effects models predicting self-reported items in participants who returned for follow-up, when accounting for effects of group, time, and their interaction

Predictor:	Group	Time	Group x Time
**Neg. Anxious**	$F(2, 284)=2.69$, $p=0.070$	$F(1, 284)=0.00$, $p=0.980$	$F(2, 284)=0.72$, $p=0.489$
**Diff. Decide**	$F(2, 284)=0.66$, $p=0.519$	$F(1, 283)=3.58$, $p=0.060$	$F(2, 283)=0.17$, $p=0.846$
**App. Reward**	$F(2, 283)=8.54$, $p<0.001$<br><br>D/A=4.815; HCs=4.239; SUDs=5.627<br>D/A - HCs: $t(284)=1.858$, $p=0.064$, $d=0.37$	$F(1, 283)=1.19$, $p=0.276$	$F(2, 284)=0.31$, $p=0.733$

	D/A - SUDs: *t*(284)=-2.78, *p*=0.006, *d*=-0.53<br>HCs - SUDs: *t*(284)=-4.068, *p*<0.001, *d*=-0.9		
**Avoid Neg.**	*F*(2, 284)=4.98, *p*=0.007<br><br>D/A=3.044; HCs=3.333; SUDs=2.386<br><br>D/A - HCs: *t*(284)=-1.005, *p*=0.316, *d*=-0.19<br>D/A - SUDs: *t*(284)=2.432, *p*=0.016, *d*=0.42<br>HCs - SUDs: *t*(284)=2.995, *p*=0.003, *d*=0.61	*F*(1, 284)=0.04, *p*=0.845	*F*(2, 283)=0.67, *p*=0.513

Note: HCs = Healthy Comparisons; D/A = Depression and Anxiety; SUDs = Substance Use Disorders; T1 = Baseline; T2 = Follow-up; NS = nonsignificant. For interpretability, sum coding was used time (baseline = −1; follow-up = 1), and group (with HCs coded as -1).

Supplementary Table 23. Results of linear mixed effects models predicting self-reported items in participants who returned for follow-up, when accounting for effects of group, time, age, and sex

Predictor:	Group	Time	Age	Sex	Group x Age	Group x Sex
**Neg. Anxious**	*F*(2, 278)=1.00, *p*=0.369	*F*(1, 291)=0.14, *p*=0.713	*F*(1, 279)=5.60, *p*=0.019	*F*(1, 278)=2.06, *p*=0.153	*F*(2, 283)=0.35, *p*=0.704	*F*(2, 278)=3.16, *p*=0.044<br><br><u>Female EMM:</u><br>D/A=4.264; HCs=4.38; SUDs=3.52<br><u>Male EMM:</u><br>D/A=3.923; HCs=3.276; SUDs=3.952<br><br><u>Female:</u><br>D/A - HCs: *t*(278.01)=-0.391, *p*=0.696, *d*=-0.08<br>D/A - SUDs: *t*(278.12)=2.73, *p*=0.007, *d*=0.54<br>HCs - SUDs: *t*(278.06)=2.607, *p*=0.010, *d*=0.63<br><u>Male:</u><br>D/A - HCs: *t*(278.03)=1.369, *p*=0.172, *d*=0.47<br>D/A - SUDs: *t*(278.27)=-0.059, *p*=0.953, *d*=-0.02<br>HCs - SUDs: *t*(278.14)=-1.33, *p*=0.185, *d*=-0.49

**Diff. Decide**	$F(2, 278)=1.43$, $p=0.241$	$F(1, 290)=3.68$, $p=0.056$	$F(1, 278)=1.02$, $p=0.313$	$F(1, 278)=0.02$, $p=0.883$	$F(2, 282)=0.70$, $p=0.499$	$F(2, 278)=1.35$, $p=0.262$
**App. Reward**	$F(2, 278)=6.70$, $p=0.001$<br><br>D/A=4.807; HCs=4.2; SUDs=5.618<br><br>D/A - HCs: $t(278)=1.942$, $p=0.053$, $d=0.4$<br>D/A - SUDs: $t(278)=-2.773$, $p=0.006$, $d=-0.53$<br>HCs - SUDs: $t(278)=-4.133$, $p<0.001$, $d=-0.92$	$F(1, 292)=1.72$, $p=0.191$	$F(1, 279)=2.61$, $p=0.107$	$F(1, 278)=0.30$, $p=0.584$	$F(2, 284)=1.11$, $p=0.332$	$F(2, 278)=0.67$, $p=0.512$
**Avoid Neg.**	$F(2, 278)=3.51$, $p=0.031$<br><br>D/A=3.056; HCs=3.378; SUDs=2.385<br><br>D/A - HCs: $t(278)=-1.103$, $p=0.271$, $d=-0.21$<br>D/A - SUDs: $t(278)=2.462$, $p=0.014$, $d=0.43$<br>HCs - SUDs: $t(278)=3.103$, $p=0.002$, $d=0.64$	$F(1, 291)=0.00$, $p=0.996$	$F(1, 279)=0.04$, $p=0.834$	$F(1, 278)=0.04$, $p=0.844$	$F(2, 283)=0.59$, $p=0.555$	$F(2, 278)=1.06$, $p=0.347$

Note: HCs = Healthy Comparisons; D/A = Depression and Anxiety; SUDs = Substance Use Disorder; T1 = Baseline; T2 = Follow-up; NS = nonsignificant.
For interpretability, age was centered, and sum coding was used for sex (female = −1; male = 1), time (baseline = −1; follow-up = 1), and group (with HCs coded as -1).

Supplementary Table 24. Results of linear mixed effects models predicting self-reported items in participants who returned for follow-up, when accounting for effects of group, time age, sex, and WRAT scores

Predictor:	Group	Time	Age	Sex	WRAT	Group x Age	Group x Sex	Group x WRAT
**Neg. Anxious**	*F*(2, 232)=2.14, *p*=0.120	*F*(1, 244)=0.10, *p*=0.757	*F*(1, 232)=2.71, *p*=0.101	*F*(1, 231)=2.09, *p*=0.150	*F*(1, 232)=0.07, *p*=0.797	*F*(2, 236)=0.86, *p*=0.424	*F*(2, 231)=2.08, *p*=0.127	*F*(2, 233)=3.37, *p*=0.036<br><br>D/A=4.17; HCs=4.108; SUDs=3.193<br><br>D/A - HCs: *t*(232)=0.217, *p*=0.828, *d*=0.05<br>D/A - SUDs: *t*(233.13)=3.345, *p*<0.001, *d*=0.72<br>HCs - SUDs: *t*(230.59)=2.718, *p*=0.007, *d*=0.68
**Diff. Decide**	*F*(2, 232)=0.48, *p*=0.620	*F*(1, 243)=1.33, *p*=0.251	*F*(1, 231)=0.16, *p*=0.692	*F*(1, 231)=0.07, *p*=0.791	*F*(1, 232)=1.19, *p*=0.276	*F*(2, 235)=0.45, *p*=0.640	*F*(2, 231)=0.81, *p*=0.447	*F*(2, 233)=0.14, *p*=0.872
**App. Reward**	*F*(2, 232)=5.64, *p*=0.004<br><br>D/A=4.926; HCs=3.966; SUDs=5.746<br><br>D/A - HCs: *t*(232.23)=2.681, *p*=0.008, *d*=0.65<br>D/A - SUDs: *t*(233.2)=-2.261, *p*=0.025, *d*=-0.56<br>HCs - SUDs: *t*(231.04)=-4.261, *p*<0.001, *d*=-1.21	*F*(1, 244)=1.13, *p*=0.289	*F*(1, 232)=5.09, *p*=0.025	*F*(1, 231)=1.08, *p*=0.300	*F*(1, 232)=0.04, *p*=0.851	*F*(2, 238)=1.63, *p*=0.198	*F*(2, 231)=0.50, *p*=0.605	*F*(2, 233)=0.47, *p*=0.626
**Avoid Neg.**	*F*(2, 232)=4.43, *p*=0.013<br><br>D/A=2.951; HCs=3.565; SUDs=2.159	*F*(1, 243)=0.05, *p*=0.826	*F*(1, 231)=1.11, *p*=0.294	*F*(1, 231)=1.26, *p*=0.262	*F*(1, 232)=0.06, *p*=0.814	*F*(2, 235)=3.46, *p*=0.033<br><br>D/A=2.951; HCs=3.565; SUDs=2.159	*F*(2, 231)=0.82, *p*=0.440	*F*(2, 233)=0.28, *p*=0.756

	D/A - HCs:<br>*t*(231.92)=-1.898,<br>*p*=0.059, *d*=-0.39<br>D/A - SUDs:<br>*t*(233.1)=2.422,<br>*p*=0.016, *d*=0.5<br>HCs - SUDs:<br>*t*(230.47)=3.732,<br>*p*<0.001, *d*=0.9					D/A - HCs:<br>*t*(231.92)=-1.898,<br>*p*=0.059, *d*=-0.39<br>D/A - SUDs:<br>*t*(233.1)=2.422,<br>*p*=0.016, *d*=0.5<br>HCs - SUDs:<br>*t*(230.47)=3.732,<br>*p*<0.001, *d*=0.9		

Note: HCs = Healthy Comparisons; D/A = Depression and Anxiety; SUDs = Substance Use Disorder; T1 = Baseline; T2 = Follow-up; NS = nonsignificant. For interpretability, age and WRAT scores were centered, and sum coding was used for sex (female = −1; male = 1), time (baseline = −1; follow-up = 1), and group (with HCs coded as -1).

Supplementary Table 25. Summary statistics for model parameters (Mean (SD)) for various sub-diagnosis at baseline and follow-up in the exploratory and the confirmatory samples

	**Baseline**		**Follow-up**		***p***
	Exploratory	***Confirmatory***	***Exploratory***	***Confirmatory***	
**Alcohol**	55	63	27	26	
**DU**	1.51 (0.90)	1.29 (1.03)	0.66 (1.22)	0.81 (1.01)	0.001
**EC**	0.35 (0.59)	0.44 (0.68)	0.34 (0.58)	0.37 (0.78)	0.88
**Cannabis**	84	74	40	29	
**DU**	1.44 (0.96)	0.92 (0.97)	1.10 (1.04)	0.47 (0.94)	<0.001
**EC**	0.56 (0.81)	0.34 (0.71)	0.61 (0.71)	0.01 (0.65)	0.002
**Stimulant**	121	157	63	72	
**DU**	1.42 (0.89)	1.17 (1.01)	0.92 (1.06)	0.73 (1.07)	<0.001
**EC**	0.48 (0.76)	0.42 (0.68)	0.48 (0.71)	0.24 (0.81)	0.132
**Opioid**	64	58	32	30	
**DU**	1.51 (0.84)	1.14 (0.98)	0.83 (1.09)	0.68 (1.00)	<0.001
**EC**	0.44 (0.67)	0.44 (0.64)	0.56 (0.73)	0.29 (0.79)	0.507
**Hallucinogen**	11	3	4	1	
**DU**	1.94 (0.31)	1.73 (0.57)	2.18 (0.57)	0.14 (NA)	NA
**EC**	0.61 (0.40)	0.34 (0.65)	0.81 (0.27)	0.37 (NA)	NA
**MDD**	315	304	216	176	
**DU**	1.27 (0.92)	1.01 (1.02)	0.69 (1.14)	0.68 (1.11)	<0.001
**EC**	0.73 (0.81)	0.68 (0.76)	0.82 (0.84)	0.60 (0.84)	0.061
**GAD**	136	141	97	88	
**DU**	1.24 (0.91)	0.97 (0.99)	0.67 (1.10)	0.48 (1.08)	<0.001
**EC**	0.81 (0.81)	0.69 (0.76)	0.87 (0.88)	0.60 (0.84)	0.096
**Social**	68	96	44	58	
**DU**	1.03 (0.92)	0.90 (0.95)	0.53 (1.07)	0.61 (1.05)	0.021

**EC**	0.77 (0.81)	0.69 (0.78)	0.67 (0.88)	0.51 (0.83)	0.345
**Panic**	73	40	48	28	
**DU**	1.37 (0.87)	0.85 (1.07)	0.80 (1.20)	0.44 (1.17)	<0.001
**EC**	0.78 (0.91)	0.63 (0.65)	0.81 (0.90)	0.48 (0.62)	0.262
**PTSD**	77	46	52	28	
**DU**	1.29 (0.90)	1.15 (1.01)	0.76 (1.18)	0.64 (1.11)	0.005
**EC**	0.81 (0.87)	0.69 (0.72)	0.89 (0.84)	0.59 (0.82)	0.384

Supplementary Table 26. Results of linear mixed effects models predicting *DU* when accounting for effects of group, time, and their interaction

Predictor:	Group	Time	Group x Time
**Alcohol**	*F*(1, 298)=9.02, *p*=0.003<br><br>HCs=0.802; Alcohol=1.171<br><br>HCs - Alcohol: *t*(278.45)=-3.365, *p*<0.001, *d*=-0.57	*F*(1, 208)=40.32, *p*<0.001<br><br>T1=1.107; T2=0.682<br><br>T1 - T2: *t*(202.21)=6.272, *p*<0.001, *d*=0.66	*F*(1, 208)=3.32, *p*=0.070
**Cannabis**	*F*(1, 333)=7.39, *p*=0.007<br><br>HCs=0.805; Cannabis=1.082<br><br>HCs - Cannabis: *t*(311.22)=-2.776, *p*=0.006, *d*=-0.41	*F*(1, 234)=21.05, *p*<0.001<br><br>T1=1.049; T2=0.731<br><br>T1 - T2: *t*(231.06)=4.675, *p*<0.001, *d*=0.47	*F*(1, 234)=0.02, *p*=0.890
**Stimulant**	*F*(1, 429)=12.91, *p*<0.001<br><br>HCs=0.805; Stimulant=1.134<br><br>HCs - Stimulant: *t*(409.96)=-3.77, *p*<0.001, *d*=-0.46	*F*(1, 311)=36.01, *p*<0.001<br><br>T1=1.138; T2=0.77<br><br>T1 - T2: *t*(321.21)=6.122, *p*<0.001, *d*=0.52	*F*(1, 311)=0.30, *p*=0.587
**Opioid**	*F*(1, 293)=9.31, *p*=0.002<br><br>HCs=0.799; Opioid=1.141<br>HCs - Opioid: *t*(278.14)=-3.293, *p*=0.001, *d*=-0.53	*F*(1, 216)=36.16, *p*<0.001<br><br>T1=1.091; T2=0.697<br>T1 - T2: *t*(211.86)=5.989, *p*<0.001, *d*=0.61	*F*(1, 216)=1.52, *p*=0.218
**Sedative**	*F*(1, 282)=4.42, *p*=0.036<br><br>HCs=0.802; Sedative=1.083<br>HCs - Sedative: *t*(257.17)=-2.341, *p*=0.020, *d*=-0.44	*F*(1, 191)=22.92, *p*<0.001<br><br>T1=1.028; T2=0.656<br><br>T1 - T2: *t*(178.76)=5.17, *p*<0.001, *d*=0.58	*F*(1, 191)=0.87, *p*=0.353
**Hallucinogen**	*F*(1, 228)=16.18, *p*<0.001<br><br>HCs=0.79; Hallucinogen=1.83<br><br>HCs - Hallucinogen:	*F*(1, 163)=1.65, *p*=0.201	*F*(1, 163)=0.20, *p*=0.655

	t(210.11)=-4.143, *p*<0.001, *d*=-1.65		
**MDD**	*F*(1, 753)=4.45, *p*=0.035<br><br>HCs=0.796; MDD=0.977<br><br>HCs - MDD: *t*(736.85)=-2.239, *p*=0.025, *d*=-0.26	*F*(1, 588)=53.19, *p*<0.001<br><br>T1=1.096; T2=0.701<br><br>T1 - T2: *t*(607.18)=9.344, *p*<0.001, *d*=0.56	*F*(1, 588)=0.73, *p*=0.393
**GAD**	*F*(1, 431)=1.49, *p*=0.222	*F*(1, 343)=55.12, *p*<0.001<br><br>T1=1.036; T2=0.63<br><br>T1 - T2: *t*(346.3)=7.979, *p*<0.001, *d*=0.62	*F*(1, 343)=1.53, *p*=0.217
**Social**	*F*(1, 320)=0.14, *p*=0.712	*F*(1, 258)=24.37, *p*<0.001<br><br>T1=0.938; T2=0.621<br><br>T1 - T2: *t*(258.69)=4.942, *p*<0.001, *d*=0.45	*F*(1, 258)=0.04, *p*=0.851
**Panic**	*F*(1, 271)=3.47, *p*=0.064	*F*(1, 219)=31.94, *p*<0.001<br><br>T1=1.034; T2=0.661<br><br>T1 - T2: *t*(218.59)=5.614, *p*<0.001, *d*=0.55	*F*(1, 219)=0.71, *p*=0.399
**PTSD**	*F*(1, 282)=4.08, *p*=0.044<br><br>HCs=0.791; PTSD=1.022<br><br>HCs - PTSD: *t*(274.13)=-2.201, *p*=0.029, *d*=-0.34	*F*(1, 227)=39.79, *p*<0.001<br><br>T1=1.059; T2=0.65<br><br>T1 - T2: *t*(226.04)=6.19, *p*<0.001, *d*=0.6	*F*(1, 227)=2.06, *p*=0.152

Note: T1 = Baseline; T2 = Follow-up; NS = nonsignificant. For interpretability, sum coding was used for time (baseline = −1; follow-up = 1), and group (with HCs coded as -1).

Supplementary Table 27. Results of linear mixed effects models predicting *DU* when accounting for effects of group, time, age, and sex

Predictor:	Group	Time	Age	Sex	Group x Age	Group x Sex
**Alcohol**	*F*(1, 271)=11.86, *p*<0.001<br><br>HCs=0.792;	*F*(1, 202)=40.11, *p*<0.001<br><br>T1=1.11;	*F*(1, 247)=0.36, *p*=0.547	*F*(1, 250)=2.28, *p*=0.133	*F*(1, 276)=2.99, *p*=0.085	*F*(1, 270)=0.64, *p*=0.425

	Alcohol=1.166<br><br>HCs - Alcohol: *t*(273.14)=-3.34, *p*<0.001, *d*=-0.58	T2=0.679<br><br>T1 - T2: *t*(202.06)=6.333, *p*<0.001, *d*=0.66				
**Cannabis**	*F*(1, 304)=8.83, *p*=0.003<br><br>HCs=0.801; Cannabis=1.075<br><br>HCs - Cannabis: *t*(306.27)=-2.739, *p*=0.007 *d*=-0.4	*F*(1, 228)=23.64, *p*< 0.001<br><br>T1=1.052; T2=0.723<br><br>T1 - T2: *t*(228.41)=4.862, *p*<0.001, *d*=0.48	*F*(1, 277)=0.22, *p*=0.636	*F*(1, 280)=2.23, *p*=0.136	*F*(1, 307)=0.74, *p*=0.390	*F*(1, 305)=1.67, *p*=0.198
**Stimulant**	*F*(1, 409)=15.92, *p*<0.001<br><br>HCs=0.815; Stimulant=1.127<br><br>HCs - Stimulant: *t*(405.97)=-3.606, *p*<0.001, *d*=-0.44	*F*(1, 320)=40.45, *p*<0.001<br><br>T1=1.142; T2=0.759<br><br>T1 - T2: *t*(320.14)=6.36, *p*<0.001, *d*=0.54	*F*(1, 378)=0.28, *p*=0.594	*F*(1, 383)=2.32, *p*=0.128	*F*(1, 409)=3.44, *p*=0.064	*F*(1, 410)=1.92, *p*=0.166
**Opioid**	*F*(1, 269)=13.35, *p*<0.001<br><br>HCs=0.795; Opioid=1.144<br><br>HCs - Opioid: *t*(271.81)=-3.366, *p*<0.001, *d*=-0.54	*F*(1, 212)=36.72, *p*<0.001<br><br>T1=1.098; T2=0.698<br><br>T1 - T2: *t*(212.93)=6.06, *p*<0.001, *d*=0.62	*F*(1, 252)=0.33, *p*=0.565	*F*(1, 255)=2.40, *p*=0.122	*F*(1, 274)=1.45, *p*=0.230	*F*(1, 274)=1.91, *p*=0.169

**Sedative**	*F*(1, 240)=9.47, *p*=0.002<br><br>HCs=0.804; Sedative=1.13 HCs - Sedative: *t*(244.11)=-2.737, *p*=0.007, *d*=-0.51	*F*(1, 176)=27.77, *p*<0.001<br><br>T1=1.045; T2=0.667<br><br>T1 - T2: *t*(176.59)=5.27, *p*<0.001, *d*=0.59	*F*(1, 215)=0.30, *p*=0.583	*F*(1, 218)=2.37, *p*=0.583	*F*(1, 248)=1.77, *p*=0.184	*F*(1, 246)=1.53, *p*=0.218
**Hallucinogen**	*F*(1, 190)=17.22, *p*<0.001<br><br>HCs=0.791; Hallucinogen=1.786<br><br>HCs - Hallucinogen: *t*(178.98)=-4.036, *p*<0.001, *d*=-1.58	*F*(1, 138)=16.97, *p*<0.001<br><br>T1=0.992; T2=0.668<br><br>T1 - T2: *t*(138.55)=4.12, *p*<0.001, *d*=0.51	*F*(1, 158)=0.24, *p*=0.623	*F*(1, 160)=2.46, *p*=0.119	*F*(1, 211)=0.00, *p*=0.946	*F*(1, 182)=0.34, *p*=0.563
**MDD**	*F*(1, 734)=4.87, *p*=0.028<br><br>HCs=0.822; MDD=0.967<br><br>HCs - MDD: *t*(724.21)=-1.825, *p*=0.068, *d*=-0.21	*F*(1, 618)=97.98, *p*<0.001<br><br>T1=1.1; T2=0.681<br><br>T1 - T2: *t*(618.71)=9.898, *p*<0.001, *d*=0.59	*F*(1, 719)=0.32, *p*=0.572	*F*(1, 725)=2.23, *p*=0.136	*F*(1, 730)=9.10, *p*=0.003<br><br>HCs=0.004; MDD=0.025<br><br>HCs - MDD: *t*(730)=-3.016, *p*=0.003, *d*=-0.03	*F*(1, 739)=1.12, *p*=0.289
**GAD**	*F*(1, 423)=1.47, *p*=0.227<br><br>HCs=0.822; GAD=0.894;<br><br>HCs - GAD: *t*(416.47)=-	*F*(1, 352)=69.52, *p*<0.001<br><br>T1=1.036; T2=0.612<br><br>T1 - T2: *t*(352.89)=8.338	*F*(1, 412)=0.35, *p*=0.552	*F*(1, 415)=2.27, *p*=0.133	*F*(1, 421)=6.52, *p*=0.011<br><br>HCs=0.004; GAD=0.025;<br><br>HCs - GAD: *t*(422)=-2.55, *p*=0.011, *d*=-0.03	*F*(1, 425)=0.53, *p*=0.467

	0.809, $p=0.419$, $d=-0.11$	, $p<0.001$, $d=0.66$				
**Social**	$F(1, 309)=1.86$, $p=0.174$	$F(1, 263)=28.39$, $p<0.001$<br><br>T1=0.959; T2=0.618<br><br>T1 - T2: $t(263.75)=5.328$, $p<0.001$, $d=0.49$	$F(1, 294)=0.25$, $p=0.614$	$F(1, 298)=2.48$, $p=0.116$	$F(1, 304)=11.66$, $p<0.001$<br><br>HCs=0.003; Social=0.034;<br><br>HCs - GAD: $t(305)=-3.41$, $p<0.001$, $d=-0.045$	$F(1, 309)=0.19$, $p=0.659$
**Panic**	$F(1, 265)=4.33$, $p=0.038$<br><br>HCs=0.805; Panic=0.968<br><br>HCs - Panic: $t(261.82)=-1.531$, $p=0.127$, $d=-0.24$	$F(1, 224)=35.23$, $p<0.001$<br><br>T1=1.027; T2=0.631<br><br>T1 - T2: $t(224.13)=5.935$, $p<0.001$, $d=0.59$	$F(1, 252)=0.31$, $p=0.576$	$F(1, 255)=2.37$, $p=0.125$	$F(1, 256)=12.45$, $p<0.001$<br><br>HCs=0.003; Panic=0.037<br><br>HCs - Panic: $t(257)=-3.53$, $p<0.001$, $d=-0.050$	$F(1, 266)=1.80$, $p=0.181$
**PTSD**	$F(1, 276)=3.38$, $p=0.067$	$F(1, 230)=40.31$, $p<0.001$<br><br>T1=1.058; T2=0.634<br><br>T1 - T2: $t(230.35)=6.349$, $p<0.001$, $d=0.62$	$F(1, 261)=0.34$, $p=0.559$	$F(1, 264)=2.33$, $p=0.128$	$F(1, 274)=3.58$, $p=0.059$	$F(1, 277)=0.07$, $p=0.794$

Note: T1 = Baseline; T2 = Follow-up; NS = nonsignificant. For interpretability, age was centered, and sum coding was used for sex (female = −1; male = 1), time (baseline = −1; follow-up = 1), and group (with HCs coded as -1).

Supplementary Table 28. Results of linear mixed effects models predicting *EC* when accounting for effects of group, time, and their interaction

Predictor:	Group	Time	Group x Time

**Alcohol**	*F*(1, 297)=43.00, *p*<0.001<br><br>HCs=1.022; Alcohol=0.421<br><br>HCs - Alcohol: *t*(279.2)=6.662, *p*<0.001, *d*=1.28	*F*(1, 200)=2.65, *p*=0.105	*F*(1, 200)=0.12, *p*=0.729
**Cannabis**	*F*(1, 335)=40.56, *p*<0.001<br><br>HCs=1.021; Cannabis=0.467<br><br>HCs - Cannabis: *t*(314.53)=6.393, *p*<0.001, *d*=1.14	*F*(1, 218)=1.56, *p*=0.213	*F*(1, 218)=0.69, *p*=0.409
**Stimulant**	*F*(1, 436)=60.93, *p*<0.001<br><br>HCs=1.021; Stimulant=0.461<br><br>HCs - Stimulant: *t*(416.83)=7.827, *p*<0.001, *d*=1.12	*F*(1, 296)=2.92, *p*=0.089	*F*(1, 296)=0.55, *p*=0.461
**Opioid**	*F*(1, 294)=37.67, *p*<0.001<br><br>HCs=1.023; Opioid=0.469<br><br>HCs - Opioid: *t*(279.17)=6.23, *p*<0.001, *d*=1.1	*F*(1, 210)=3.07, *p*=0.081	*F*(1, 210)=0.04, *p*=0.839
**Sedative**	*F*(1, 279)=37.90, *p*<0.001<br><br>HCs=1.022; Sedative=0.358<br><br>HCs - Sedative: *t*(256.41)=6.427, *p*<0.001, *d*=1.38	*F*(1, 181)=4.21, *p*=0.042<br><br>T1=0.778; T2=0.899<br><br>T1 - T2: *t*(171.43)=-2.209, *p*=0.029, *d*=-0.25	*F*(1, 181)=0.19, *p*=0.663
**Hallucinogen**	*F*(1, 219)=2.25, *p*=0.135	*F*(1, 147)=1.34, *p*=0.249	*F*(1, 147)=0.20, *p*=0.653
**MDD**	*F*(1, 759)=20.53, *p*<0.001<br><br>HCs=1.024; MDD=0.727<br><br>HCs - MDD: *t*(742.95)=4.492, *p*<0.001, *d*=0.56	*F*(1, 577)=4.40, *p*=0.036<br><br>T1=0.764; T2=0.829<br><br>T1 - T2: *t*(592.98)=-2.059, *p*=0.04, *d*=-0.12	*F*(1, 577)=0.43, *p*=0.510
**GAD**	*F*(1, 434)=12.51, *p*<0.001	*F*(1, 333)=2.33, *p*=0.128	*F*(1, 333)=1.24, *p*=0.267

	HCs=1.025; GAD=0.759<br><br>HCs - GAD: *t*(425.25)=3.463, *p*<0.001, *d*=0.55		
**Social**	*F*(1, 324)=15.56, *p*<0.001<br><br>HCs=1.035; Social=0.692<br><br>HCs - Social: *t*(324.47)=3.945, *p*<0.001, *d*=0.66	*F*(1, 246)=0.17, *p*=0.679	*F*(1, 246)=3.10, *p*=0.079
**Panic**	*F*(1, 272)=9.33, *p*=0.002<br><br>HCs=1.026; Panic=0.733<br><br>HCs - Panic: *t*(266.47)=3.003, *p*=0.003, *d*=0.58	*F*(1, 212)=1.33, *p*=0.249	*F*(1, 212)=0.78, *p*=0.378
**PTSD**	*F*(1, 283)=8.03, *p*=0.005<br><br>HCs=1.026; PTSD=0.767<br><br>HCs - PTSD: *t*(275.73)=2.776, *p*=0.006, *d*=0.48	*F*(1, 221)=1.20, *p*=0.275	*F*(1, 221)=0.77, *p*=0.380

Note: T1 = Baseline; T2 = Follow-up; NS = nonsignificant. For interpretability, sum coding was used for time (baseline = −1; follow-up = 1), and group (with HCs coded as -1).

Supplementary Table 29. Results of linear mixed effects models predicting *EC* when accounting for effects of group, time, age, and sex

Predictor:	Group	Time	Age	Sex	Group x Age	Group x Sex
**Alcohol**	*F*(1, 271)=39.10, *p*<0.001<br><br>HCs=0.979; Alcohol=0.408<br><br>HCs - Alcohol: *t*(272.8)=6.345, *p*<0.001, *d*=1.22	*F*(1, 196)=3.11, *p*=0.079	*F*(1, 251)=0.23, *p*=0.630	*F*(1, 253)=20.59, *p*<0.001<br><br>Female=0.82; Male=0.587<br><br>Female - Male: *t*(270.53)=2.569, *p*=0.011, *d*=0.5	*F*(1, 276)=1.22, *p*=0.270	*F*(1, 270)=9.57, *p*=0.002<br><br>Female EMM:<br>HCs=1.235; Alcohol=0.385<br><br>Male EMM:<br>HCs=0.721; Alcohol =0.432<br><br>Female:<br>HCs - Alcohol: *t*(268.68)=6.375, *p*<0.001, *d*=1.81

						Male:<br>HCs - Alcohol:<br>*t*(274.97)=2.374,<br>*p*=0.018, *d*=0.62
**Cannabis**	*F*(1, 306)=33.98, *p*<0.001<br><br>HCs=1.009; Cannabis=0.458<br><br>HCs - Cannabis: *t*(307.77)=6.554, *p*<0.001, *d*=1.13	*F*(1, 217)=2.25, *p*=0.135	*F*(1, 286)=0.19,*p*=0.662	*F*(1, 288)=19.00, *p*<0.001<br><br>Female=0.828; Male=0.672<br><br>Female - Male: *t*(303.99)=1.852, *p*=0.065, *d*=0.32	*F*(1, 310)=0.34, *p*=0.558	*F*(1, 306)=21.71, *p*<0.001<br><br>Female EMM:<br>HCs=1.236; Cannabis=0.338<br>Male EMM:<br>HCs=0.722; Cannabis=0.611<br><br>Female:<br>HCs - Cannabis:<br>*t*(300.15)=7.873, *p*<0.001, *d*=1.85<br>Male:<br>HCs - Cannabis:<br>*t*(315.18)=0.899, *p*=0.369, *d*=0.23
**Stimulant**	*F*(1, 414)=47.40, *p*<0.001<br><br>HCs=1.032; Stimulant=0.459<br><br>HCs - Stimulant: *t*(411.81)=8.18, *p*<0.001, *d*=1.15	*F*(1, 305)=2.59, *p*=0.108	*F*(1, 390)=0.23, *p*=0.628	*F*(1, 393)=21.16, *p*<0.001<br><br>Female=0.726; Male=0.628<br><br>Female - Male: *t*(428.22)=1.413, *p*=0.158, *d*=0.2	*F*(1, 416)=1.33, *p*=0.250	*F*(1, 415)=23.43, *p*<0.001<br><br>Female EMM:<br>HCs=1.235; Stimulant=0.39<br>Male EMM:<br>HCs=0.722; Stimulant=0.566<br><br>Female:<br>HCs - Stimulant:<br>*t*(400.28)=9.268, *p*<0.001, *d*=1.69<br>Male:<br>HCs - Stimulant:<br>*t*(427.94)=1.428, *p*=0.154, *d*=0.31

**Opioid**	$F(1, 270)=33.22$, $p<0.001$<br><br>HCs=1.009; Opioid=0.461<br><br>HCs - Opioid: $t(272.24)=6.325$, $p<0.001$, $d=1.09$	$F(1, 209)=3.54$, $p=0.061$	$F(1, 255)=0.27$, $p=0.605$	$F(1, 257)=20.40$, $p<0.001$<br><br>Female=0.9; Male=0.649<br><br>Female - Male: $t(269.11)=2.897$, $p=0.004$, $d=0.5$	$F(1, 275)=0.22$, $p=0.638$	$F(1, 274)=13.73$, $p<0.001$<br><br>Female EMM:<br>HCs=1.237; Opioid=0.4<br>Male EMM:<br>HCs=0.725; Opioid=0.537<br><br>Female<br>HCs - Opioid: $t(264.93)=6.995$, $p<0.001$, $d=1.67$<br>Male:<br>HCs - Opioid: $t(282.7)=1.485$, $p=0.139$, $d=0.37$
**Sedative**	$F(1, 239)=32.02$, $p<0.001$<br><br>HCs=1.028; Sedative=0.356<br><br>HCs - Sedative: $t(242.83)=6.742$, $p<0.001$, $d=1.4$	$F(1, 171)=4.52$, $p=0.035$<br><br>T1=0.781; T2=0.897<br><br>T1 - T2: $t(171.9)=-2.126$, $p=0.035$, $d=-0.24$	$F(1, 219)=0.28$, $p=0.598$	$F(1, 221)=19.94$, $p<0.001$<br><br>Female=0.967; Male=0.626<br><br>Female - Male: $t(230.47)=3.629$, $p<0.001$, $d=0.71$	$F(1, 247)=0.28$, $p=0.595$	$F(1, 244)=8.20$, $p=0.005$<br><br>Female EMM:<br>HCs=1.238; Sedative=0.331<br>Male EMM:<br>HCs=0.725; Sedative=0.391<br><br>Female:<br>HCs - Sedative: $t(237.21)=6.97$, $p<0.001$, $d=1.89$<br>Male:<br>HCs - Sedative: $t(250.48)=2.18$, $p=0.030$, $d=0.7$
**Hallucinogen**	$F(1, 183)=2.89$, $p=0.091$	$F(1, 134)=3.37$, $p=0.069$	$F(1, 160)=0.23$, $p=0.634$	$F(1, 161)=17.81$, $p<0.001$<br><br>Female=1.196; Male=0.725<br><br>Female - Male: $t(162.93)=4.026$, $p<0.001$, $d=0.99$	$F(1, 198)=0.08$, $p=0.773$	$F(1, 177)=2.09$, $p=0.150$

**MDD**	*F*(1, 741)=16.50, *p*<0.001<br><br>HCs=1.067; MDD=0.723<br><br>HCs - MDD: *t*(732.88)=5.147, *p*<0.001, *d*=0.65	*F*(1, 602)=3.32, *p*=0.069	*F*(1, 731)=0.20, *p*=0.658	*F*(1, 734)=18.96, *p*<0.001<br><br>Female=0.864; Male=0.656<br><br>Female - Male: *t*(776)=3.701, *p* < 0.001, *d*=0.39	*F*(1, 740)=2.13, *p*=0.145	*F*(1, 745)=8.34, *p*=0.004<br><br>Female EMM: HCs=1.235; MDD=0.765<br>Male EMM: HCs=0.722; MDD=0.639<br><br>Female: HCs - MDD: *t*(727.08)=5.553, *p*<0.001, *d*=0.89<br>Male: HCs - MDD: *t*(754.87)=0.798, *p*=0.425, *d*=0.16
**GAD**	*F*(1, 425)=9.67, *p*=0.002<br><br>HCs=1.086; GAD=0.746<br><br>HCs - GAD: *t*(419.61)=4.393, *p*<0.001, *d*=0.7	*F*(1, 344)=1.20, *p*=0.274	*F*(1, 417)=0.13, *p*=0.720	*F*(1, 418)=17.96, *p*<0.001<br><br>Female=0.953; Male=0.677<br><br>Female - Male: *t*(431.62)=3.399, *p*<0.001, *d*=0.57	*F*(1, 426)=2.63, *p*=0.106	*F*(1, 426)=5.45, *p*=0.020<br><br>Female EMM: HCs=1.238; GAD=0.786;<br>Male EMM: HCs=0.723; GAD=0.65<br><br>Female: HCs - GAD: *t*(415.86)=4.778, *p*<0.001, *d*=0.93<br>Male: HCs - GAD: *t*(431.66)=0.549, *p*=0.584, *d*=0.15
**Social**	*F*(1, 312)=9.38, *p*=0.002	*F*(1, 252)=0.10, *p*=0.754	*F*(1, 301)=0.13,	*F*(1, 303)=18.53, *p*<0.001	*F*(1, 310)=3.24,	*F*(1, 312)=2.51, *p*=0.114

	HCs=0.985; Social=0.677 HCs - Social: *t*(313.89)=3.584, *p*<0.001, *d*=0.59		*p*=0.722	Female=1.022; Male=0.641<br><br>Female - Male: *t*(312.12)=4.42, *p*<0.001, *d*=0.73	*p*=0.073	
**Panic**	*F*(1, 265)=6.41, *p*=0.012<br><br>HCs=1.056; Panic=0.718<br><br>HCs - Panic: *t*(263.07)=3.513, *p*<0.001, *d*=0.67	*F*(1, 216)=1.51, *p*=0.220	*F*(1, 257)=0.16, *p*=0.693	*F*(1, 258)=17.41, *p*<0.001<br><br>Female=1.033; Male=0.708<br><br>Female - Male: *t*(264.28)=3.297, *p*=0.001, *d*=0.65	*F*(1, 261)=2.65, *p*=0.104	*F*(1, 266)=5.16, *p*=0.024<br><br><u>Female EMM:</u> HCs=1.238; Panic=0.736; <u>Male EMM:</u> HCs=0.724; Panic=0.685<br><br><u>Female:</u> HCs - Panic: *t*(258.8)=4.304, *p*<0.001, *d*=1 <u>Male:</u> HCs - Panic: *t*(270.01)=0.234, *p*=0.815, *d*=0.08
**PTSD**	*F*(1, 276)=7.24, *p*=0.008<br><br>HCs=1.052; PTSD=0.741<br><br>HCs - PTSD: *t*(273.18)=3.384, *p*<0.001, *d*=0.58	*F*(1, 225)=1.23, *p*=0.268	*F*(1, 263)=0.19, *p*=0.667	*F*(1, 266)=18.17, *p*<0.001<br><br>Female=1.059; Male=0.672<br><br>Female - Male: *t*(275.13)=4.093, *p*<0.001, *d*=0.72	*F*(1, 276)=2.78, *p*=0.096	*F*(1, 277)=2.32, *p*=0.129

Note: T1 = Baseline; T2 = Follow-up; NS = nonsignificant. For interpretability, age was centered, and sum coding was used for sex (female = −1; male = 1), time (baseline = −1; follow-up = 1), and group (with HCs coded as -1).

Supplementary Table 30. Simulation-based post-hoc power analyses to detect group differences between HCs and clinicals subgroups

Group	*N at baseline (% attrition at follow-up)*	*DU*		*EC*	
		Cohen's d	*Power*	*Cohen's d*	*Power*
MDD	*619 (36.19%)*	*0.183*	*0.955*	*-0.303*	*1.000*
GAD	*277 (32.13%)*	*0.115*	*0.584*	*-0.271*	*1.000*
Social	*164 (37.80%)*	*0.039*	*0.086*	*-0.353*	*1.000*
Panic	*113 (32.74%)*	*0.199*	*0.846*	*-0.310*	*1.000*
PTSD	*123 (34.96%)*	*0.243*	*0.955*	*-0.245*	*0.977*
Alcohol	*118 (55.08%)*	*0.332*	*0.998*	*-0.631*	*1.000*
Cannabis	*158 (56.33%)*	*0.284*	*0.987*	*-0.595*	*1.000*
Stimulant	*278 (51.44%)*	*0.322*	*0.999*	*-0.599*	*1.000*
Opioid	*122 (49.18%)*	*0.317*	*0.997*	*-0.572*	*1.000*
Sedative	*84 (61.90%)*	*0.281*	*0.919*	*-0.671*	*1.000*

Supplementary Table 31. Performance metrics for predictive categorization differentiating individuals with affective disorders (no comorbid SUDs) and those with SUDs (but no comorbid affective disorders)

Classification Predictors	Method	AUC	Balanced Accuracy	Sensitivity	Specificity
**Age, Sex**	ENET	0.495	0.477	0.300	0.653
**Age, Sex**	KNN	0.600	0.569	0.600	0.538
**Age, Sex**	ADABAG	0.565	0.551	0.740	0.363
**Age, Sex**	STACK	0.617	0.551	0.720	0.381
**Model parameters***	ENET	0.752	0.648	0.609	0.688
**Model parameters**	KNN	0.620	0.596	0.607	0.584
**Model parameters**	ADABAG	0.675	0.584	0.505	0.663
**Model parameters**	STACK	0.699	0.647	0.587	0.706
**Model parameters, Age, Sex**	ENET	0.763	0.694	0.731	0.656
**Model parameters, Age, Sex**	KNN	0.670	0.632	0.711	0.553
**Model parameters, Age, Sex**	ADABAG	0.692	0.658	0.569	0.747
**Model parameters, Age, Sex**	STACK	0.740	0.704	0.707	0.700
**All parameters***	ENET	0.720	0.673	0.680	0.666
**All parameters**	KNN	0.549	0.530	0.560	0.500
**All parameters**	ADABAG	0.662	0.577	0.360	0.794
**All parameters**	STACK	0.668	0.571	0.420	0.722
**All parameters, Age, Sex**	**ENET**	**0.773**	**0.714**	**0.762**	**0.666**
**All parameters, Age, Sex**	KNN	0.573	0.553	0.604	0.503

**All parameters, Age, Sex**	ADABAG	0.668	0.629	0.545	0.713
**All parameters, Age, Sex**	STACK	0.700	0.626	0.565	0.688

***Note:** Model parameters = *EC* and *DU* at baseline and follow-up; All parameters = *EC* and *DU* (at baseline and follow-up) and *choice uncertainty*-RT correlation coefficients. The model with the highest balanced accuracy is bolded.

Supplementary Table 32. Performance metrics for predictive categorization differentiating individuals with and without SUDs

Classification Predictors	Method	AUC	Balanced Accuracy	Sensitivity	Specificity
**Age, Sex**	ENET	0.537	0.495	0.406	0.583
**Age, Sex**	KNN	0.606	0.572	0.642	0.502
**Age, Sex**	ADABAG	0.603	0.552	0.704	0.400
**Age, Sex**	Stack	0.605	0.583	0.728	0.437
**Model parameters***	ENET	0.680	0.620	0.612	0.628
**Model parameters**	KNN	0.582	0.585	0.587	0.582
**Model parameters**	ADABAG	0.664	0.621	0.592	0.649
**Model parameters**	Stack	0.658	0.622	0.605	0.640
**Model parameters, Age, Sex**	ENET	0.719	0.677	0.713	0.640
**Model parameters, Age, Sex**	KNN	0.692	0.655	0.736	0.573
**Model parameters, Age, Sex**	ADABAG	0.682	0.639	0.683	0.595
**Model parameters, Age, Sex**	Stack	0.713	0.657	0.712	0.601
**All parameters***	**ENET**	**0.761**	**0.694**	**0.653**	**0.734**
**All parameters**	KNN	0.669	0.623	0.605	0.642
**All parameters**	ADABAG	0.722	0.660	0.773	0.548
**All parameters**	Stack	0.735	0.673	0.713	0.633
**All parameters, Age, Sex**	ENET	0.753	0.684	0.683	0.684
**All parameters, Age, Sex**	KNN	0.656	0.628	0.689	0.567
**All parameters, Age, Sex**	ADABAG	0.694	0.632	0.641	0.622
**All parameters, Age, Sex**	Stack	0.722	0.691	0.719	0.662

***Note:** Model parameters = *EC* and *DU* at baseline and follow-up; All parameters = *EC* and *DU* (at baseline and follow-up) and *choice uncertainty*-RT correlation coefficients. The model with the highest balanced accuracy is bolded.

Supplementary Table 33. Performance metrics for predictive categorization differentiating individuals with and without affective disorders

Classification Predictors	Method	AUC	Balanced Accuracy	Sensitivity	Specificity
**Age, Sex**	ENET	0.447	0.473	0.352	0.594
**Age, Sex**	KNN	0.443	0.492	0.511	0.473
**Age, Sex**	ADABAG	0.519	0.507	0.844	0.170
**Age, Sex**	Stack	0.456	0.467	0.721	0.213
**Model parameters***	**ENET**	**0.748**	**0.688**	**0.756**	**0.619**
**Model parameters**	KNN	0.627	0.623	0.674	0.571
**Model parameters**	ADABAG	0.661	0.595	0.468	0.722
**Model parameters**	Stack	0.714	0.622	0.520	0.725
**Model parameters, Age, Sex**	ENET	0.625	0.610	0.624	0.596
**Model parameters, Age, Sex**	KNN	0.506	0.511	0.476	0.546
**Model parameters, Age, Sex**	ADABAG	0.617	0.539	0.406	0.672
**Model parameters, Age, Sex**	STACK	0.592	0.524	0.389	0.658
**All parameters***	ENET	0.630	0.596	0.561	0.632
**All parameters**	KNN	0.477	0.448	0.441	0.455
**All parameters**	ADABAG	0.526	0.508	0.274	0.743
**All parameters**	STACK	0.543	0.509	0.309	0.708
**All parameters, Age, Sex**	ENET	0.629	0.575	0.533	0.616
**All parameters, Age, Sex**	KNN	0.531	0.533	0.517	0.549
**All parameters, Age, Sex**	ADABAG	0.579	0.533	0.633	0.432
**All parameters, Age, Sex**	STACK	0.570	0.563	0.583	0.543

***Note:** Model parameters = *EC* and *DU* at baseline and follow-up; All parameters = *EC* and *DU* (at baseline and follow-up) and *choice uncertainty*-RT correlation coefficients. The model with the highest balanced accuracy is bolded.

Supplementary Table 34. Performance metrics for predictive categorization differentiating individuals with and without comorbid affective and substance use disorders

Classification Predictors	Method	AUC	Balanced Accuracy	Sensitivity	Specificity
**Age, Sex**	ENET	0.592	0.559	0.377	0.741
**Age, Sex**	KNN	0.539	0.523	0.580	0.465
**Age, Sex**	ADABAG	0.573	0.560	0.836	0.284
**Age, Sex**	STACK	0.551	0.580	0.828	0.332
**Model parameters***	ENET	0.640	0.599	0.653	0.546
**Model parameters**	KNN	0.587	0.562	0.581	0.543
**Model parameters**	ADABAG	0.614	0.588	0.716	0.459
**Model parameters**	STACK	0.626	0.610	0.741	0.478
**Model parameters, Age, Sex**	ENET	0.642	0.607	0.627	0.588
**Model parameters, Age, Sex**	KNN	0.655	0.618	0.718	0.518
**Model parameters, Age, Sex**	ADABAG	0.650	0.606	0.735	0.477
**Model parameters, Age, Sex**	STACK	0.675	0.610	0.743	0.477
**All parameters***	ENET	0.663	0.635	0.618	0.652
**All parameters**	KNN	0.598	0.601	0.601	0.601
**All parameters**	ADABAG	0.654	0.601	0.797	0.405
**All parameters**	STACK	0.665	0.614	0.756	0.472
**All parameters, Age, Sex**	**ENET**	**0.684**	**0.643**	**0.658**	**0.627**
**All parameters, Age, Sex**	KNN	0.627	0.603	0.667	0.538
**All parameters, Age, Sex**	ADABAG	0.661	0.600	0.652	0.549
**All parameters, Age, Sex**	STACK	0.671	0.597	0.627	0.568

***Note:** Model parameters = *EC* and *DU* at baseline and follow-up; All parameters = *EC* and *DU* (at baseline and follow-up) and *choice uncertainty*-RT correlation coefficients. The model with the highest balanced accuracy is bolded.

References

1. Smith, R., et al., *Elevated decision uncertainty and reduced avoidance drives in depression, anxiety and substance use disorders during approach–avoidance conflict: a replication study.* Journal of Psychiatry and Neuroscience, 2023. **48**(3): p. E217-E231.
